# The Distinction of Chemical Profiles of Mountainous Forest Cultivated Ginseng and Garden Ginseng Based on Calcium Oxalate Crystals, Organic Acids, and Ginsenosides

**DOI:** 10.3390/foods14173073

**Published:** 2025-08-30

**Authors:** Xiaotong Zhang, Xiaoku Ran, Yidan Xi, Deqiang Dou

**Affiliations:** College of Pharmacy, Liaoning University of Traditional Chinese Medicine, Dalian 116600, China; zhangxxxt@163.com (X.Z.); xkran@163.com (X.R.); xy_idan@163.com (Y.X.)

**Keywords:** mountainous forest cultivated ginseng, HPLC, GC/TQ, oxalic acid, organic acid, ginsenoside, chemometric analysis

## Abstract

This study aims to further analyze the chemical characteristics of mountainous forest cultivated ginseng (MFCG) and garden ginseng (GG), concerning their calcium oxalate crystals, organic acids, and ginsenosides. The results demonstrate that MFCG had higher levels of non-free oxalate, calcium oxalate crystals, and most ginsenosides, while GG had higher fumaric acid/total organic acids. The content of non-free oxalate and calcium oxalate crystals in rhizome was the highest, showing a positive correlation with the growth years (5–20 years). In most cases, in MFCG, non-free oxalic acid ≥ 0.8%, calcium oxalate ≥ 160/mg, fumaric acid/total organic acids < 9%, Rb1 ≥ 6 mg/g, PPD/PPT was close to 2, and Rb1/Ro ≥ 2.5, while in GG, non-free oxalic acid < 0.8%, calcium oxalate ≤ 60/mg, fumaric acid/total organic acids ≥ 9%, Rb1 < 6 mg/g, PPD/PPT was close to 1, and Rb1/Ro < 2.5. These results can be used as the basis for distinguishing between GG and MFCG. Chemometric analysis of non-free oxalate, calcium oxalate crystals, and ginsenosides could distinguish MFCG from GG. Chemometric analysis of succinate, citrate, and malonic acids could mostly differentiate MFCG of over 15 years from that of less than 12 years. As far as we know, the present study is the first to determine the difference in the ratio of ginsenosides (Rb1/Ro, PPD/PPT) and the ratio of organic acids, which provides an innovative method for the distinction between the two and a scientific basis for effective quality control of MFCG.

## 1. Introduction

Ginseng, the root and rhizome of Panax ginseng C. A. Mey, is a globally renowned herbal medicine and has attracted considerable interest in cultivation in China, attributed to its high economic and medicinal value. The history of ginseng stretches back thousands of years, playing a pivotal role in traditional Chinese medicine. According to the cultivation methods and growth environment, two types of ginseng medicines are available, namely garden ginseng (GG) and mountain forest cultivated ginseng (MFCG), which are described in the Chinese Pharmacopeia. The GG originates from Chinese ancient garden cultivation practices that have been passed down for over 400 years in folk medicine. Recently, the predominant cultivation method of GG is artificially on mountainsides or in shaded greenhouses in farmland, which have replaced ancient methods. MFCG thrives in its natural habitat of mountain forests, and is similar to wild ginseng in both its cultivation environment and appearance. GG is typically harvested after a growth period of 4–6 years, whereas MFCG is allowed to mature for approximately over 15 years in a wild-simulated environment, resulting in slower growth rates. Traditionally, MFCG is reputed to be more potent in comparison to GG, and its price is much more expensive than GG, owing to its unique growth environment and extended growth cycle, as people think the older it is, the better quality of ginseng it is. In the current market for MFCG products is adulterant with GG, particularly in its powder product, which makes it even harder to distinguish and identify its origin. This kind of adulteration has had a serious negative impact on the market and has become a major variable in the business and economic fields [1]. Therefore, elucidating the chemical disparities and establishing prompt and precise differentiation between GG and MFCG is imperative for upholding the authenticity and implementing stringent quality control measures for MFCG.

In recent studies, researchers have employed HPLC-DAD-ELSD analysis to identify the presence of ginsenosides in both GG and MFCG. However, when this method is applied, it can only distinguish the two locally, lacking the sensitivity and specificity for a complete distinction between them [2]. Before this, some scholars pointed out that the stable isotope analysis method could effectively distinguish between the two variants and their extracts, but due to the insufficiency of equipment, the applicability of this method was limited [3]. Furthermore, terahertz spectroscopy has been used to distinguish MFCG from GG, but this method is also limited by equipment [4]. Advanced and highly accurate technologies are currently being employed to verify the authenticity of food and pharmaceutical products [5,6]. However, compared with the above methods, such as the stable isotope method, the method adopted in this study takes the characteristic chemical components or active components in ginseng as the determination indicators, which is more rapid and simple, and the accuracy of differentiating MFCG and GG is guaranteed to a certain extent.

In addition to the main active component ginsenoside, organic acids are key metabolites mediating physiological processes from growth regulation to environmental adaptation in plants like ginseng, with demonstrated impacts on secondary metabolite biosynthesis [7,8,9,10,11,12]. Similarly, oxalic acid is a low-molecular-weight dicarboxylic acid organic acid that plays an important regulatory role in the growth and development of plants [13]. At the same time, when plants are attacked by animals or the environment, oxalate crystals will accumulate in the affected areas [14]. The stems of ginseng also accumulate oxalate crystals every year when they are newly grown. Environmental conditions and crop cultivation practices can affect the accumulation of substances in plants [15,16]. Different planting methods and cultivation environments are hypothesized to induce distinct oxalic acid, calcium oxalate crystals, organic acids, and ginsenosides between MFCG and GG, and these component differences could be applied to distinguish the two types of ginseng. However, limited research exists on the differences in these components between MFCG and GG. In this study, the determination of calcium oxalate crystals, oxalic acid, organic acids, and ginsenosides established a method to distinguish GG and MFCG, and could establish a scientific foundation for effective quality control measures of MFCG.

## 2. Materials and Methods

### 2.1. Reagents and Materials

A total of 132 batches of MFCG and GG products were collected from Liaoning and Jilin provinces, China. Analysis of oxalic acid in the whole ginseng, the samples of ginseng included 40 batches of MFCG (S1~S40) and 10 batches of GG (S41~S50). Analysis of oxalic acid in different parts of ginseng, the samples of ginseng included 12 batches of MFCG (S51~S62) and 3 batches of GG (S63~S65). Analysis of organic acids in the whole ginseng, the samples of ginseng included 17 batches of MFCG (S66~S82) and 6 batches of GG (S83~S88). Analysis of organic acids in different parts of ginseng, the samples of ginseng included 9 batches of MFCG (S89~S97) and 5 batches of GG (S98~S102). Analysis of ginsenosides in the whole ginseng, the samples of ginseng included 12 batches of MFCG (S103~S114) and 7 batches of GG (S115~S121). Analysis of ginsenosides in different parts of ginseng, the samples of ginseng included 8 batches of MFCG (S122~S129) and 3 batches of GG (S130~S132). All samples were identified by Professor Deqiang Dou of Liaoning University of Traditional Chinese Medicine, China. A detailed sample list is given in the Supplementary Material (Table S1).

Oxalic acid standard (batch number: 101097-201702, purity > 99.1%) was purchased from the National Institute for Food and Drug Control (Beijing, China). Dl-malic acid standard, malonic acid standard, citric acid standard and adipic acid standard (Sigma-Aldrich, St. Louis, MO, USA; Succinic acid standard (Sigma-Aldrich, St. Louis, MO, USA); Cinnamic acid standard, Fumaric acid standard and palmitic acid standard (Yuanye, Shanghai, China); Phosphoric acid (Quanrui reagent, Shenyang, China); Ginsenoside Rg1, Re, Rb1, Ro, Rc Rb2, Rd (National institutes for food and drug control, China) Acetonitrile HPLC grade (TEDIA, Anqing, China). Methanol HPLC grade (J.T.Baker, Dongguan, China); all other chemicals and solvents were of an analytical grade; Agilent 1260 HPLC (Agilent Technologies, Santa Clara, CA, USA); 1/10,000 analysis balance (Acculab-Sartorius Group, Gottingen, Germany); 1/100,000 analytical balance (CP225DSartorius group, Gottingen, Germany); Agilent 8890 GC system-7000E GC/TQ (Agilent Technologies, Santa Clara, CA, USA).

### 2.2. Methods

#### 2.2.1. Standard Solutions and Sample Solutions Preparation for HPLC Determination of Non-Free Oxalic Acid

Reference compounds of oxalic acid were weighed accurately (15.03 mg) and dissolved in ultrapure water, which was added into a volumetric flask (50 mL), and stored at 4 °C until the time of analysis.

Accurately weighed pulverized samples (60 mesh, 0.15 g) were combined with 15 volumes of ultrapure water and incubated at 70 °C for 10 min. The mixture was then centrifuged at 3500 r/min for 10 min and removed the supernatant. The residue was subsequently washed twice with 15 volumes of hot water (70 °C). After the free oxalic acid was removed, the residual powder acid was subjected to ultrasonic extraction using 15 volumes of 0.1 mol/L HCl, repeated three times, each for 10 min. The supernatant was obtained by centrifugation at 3500 r/min for 10 min. The supernatant was combined 3 times, and the volume of 0.1 mol/L HCl was fixed to 10 mL to determine the non-free oxalic acid peak area.

#### 2.2.2. Sample Solutions Preparation for Microscopic Identification

Pulverized samples (60 mesh, 20 mg) were accurately weighed, and 1 mL of chloral hydrate solution was added. The mixture was gently shaken to ensure thorough mixing. Subsequently, 0.02 mL of the resulting suspension was measured and heated using an alcohol lamp. An additional 0.01 mL of chloral hydrate solution was added to permeate the sample, followed by the addition of 0.015 mL of dilute glycerin solution to further permeate the sample. The prepared sample was then mounted on a microscope slide (25.4 × 76.2 mm) for microscopic observation. A cover slide (20 × 20 mm) was placed over the sample. The sample was scanned and observed under the microscope using the zigzag moving method. Calcium oxalate cluster crystals were counted as the primary microscopic features.

#### 2.2.3. Standard Solutions and Sample Solutions Preparation for GC-MS/MS

Reference compounds of adipic acid were weighed accurately (0.1 g) and dissolved in methanol, which was added into a volumetric flask (5 mL), and stored at 4 °C as the internal standard solution.

Reference compounds of oxalic acid, malic acid, citric acid, malonic acid, succinic acid, cinnamic acid, fumaric acid, and palmitic acid of different qualities and dissolved in methanol, which was added into a volumetric flask (10 mL), and stored at 4 °C until time of analysis.

Pulverized samples (60 mesh, 0.1 g) were accurately weighed, added 5 mL 5% sulfuric acid-methanol (volume fraction) solution and 20 μL adipic acid internal standard solution, reflux in a water bath at 60 °C for 2 h, and cooled to room temperature. The supernatant was taken into the separating funnel and mixed with 10 mL of distilled water, then extracted with 5 mL of dichloromethane each time, extracted 3 times, and combined with the extraction liquid, after drying, the dichloromethane was fixed to 1 mL and 0.1 g of anhydrous sodium sulfate was added to dry overnight, and the samples were injected for GC-MS/MS analysis.

1 mL of organic acids mixed in standard solution with different concentrations was put into a flask, processed according to the preparation method of sample solution, and injected into the sample for GC-MS/MS analysis. According to the ratio of the peak area of the organic acid standard to the internal standard, and the concentration ratio of each organic acid standard to the internal standard, the internal standard method was used for quantification.

#### 2.2.4. Standard Solutions and Sample Solutions Preparation for HPLC Determination of Ginsenosides

Accurately weigh the ginsenoside Rg1, Re, Rb1, Ro, Rc, Rb2, Rd reference substance; add methanol to make a mixed solution containing 0.2 mg/mL and store at 4 °C until the time of analysis.

Pulverized samples (60 mesh, 0.5 g) were accurately weighed, place it in a conical flask, add 10 mL of 70% methanol, weigh it, ultrasonically extract for 30 min, cool it to room temperature, then make up for the lost weight with 70% methanol, filter it, and take the filtrate to obtain the sample solutions.

#### 2.2.5. HPLC Conditions

HPLC of non-free oxalic acid was performed on an Agilent ZORBAX SB-Aq C18 column (4.6 × 250 mm, 5 μm), using separate mobile phases of 0.5% (by volume) phosphoric acid in water. With a flow rate of 0.8 mL/min, the column temperature was at 28 °C, the UV wavelength used for detection was set at 210 nm in a DAD detector and the injection volume was 10 μL.

HPLC of ginsenosides was performed on an Agilent InfinityLab Poroshell HPH column (3.0 × 150 mm, 2.7 μm), using separate mobile phases of (A) 0.1% (by volume) phosphoric acid in water and (B) acetonitrile. The gradient programs were optimized as: 0–14 min, 80% (A); 14–16 min, 80–70% (A), 16–28 min, 70–70% (A), 28–42 min, 70–67% (A), 42–55 min, 67–95% (A). The flow rate was 0.8 mL/min, and the column temperature was 30 °C. The UV wavelength used for detection was set at 203 nm in a DAD detector and the injection volume was 10 μL.

#### 2.2.6. Chromatographic and Mass Spectrometry Conditions of GC-MS/MS

Agilent HP-5 capillary column (30 m × 0.32 mm, 0.25 μm); The carrier gas was helium, and the inlet temperature was 280 °C. The initial temperature was increased to 40 °C for 3 min, then increased to 156 °C at 4 °C/min for 2 min, and increased to 230 °C at 10 °C/min for 3 min. Rise from 10 °C/min to 280 °C for 2 min; Shunt sampling, shunt ratio 10:1; Sample size 1 μL; The carrier gas is helium; Flow rate: 1 mL/min.

EI source, ionization energy 70 ev, temperature 230 °C; Transmission line temperature 280 °C; Four stage rod temperature 150 °C; Transmitting current 100 mA; Detection voltage 1.1 kV; Transmission line temperature 280 °C; The scanning mode is full SCAN and MRM (Table 1), and the scanning quality ranges from 40 to 450 Da. The solvent delay time was 4 min.

#### 2.2.7. Data Analysis

An external standard was utilized for the quantitative determination of non-free oxalic acid and ginsenosides. An internal standard was utilized for the quantitative determination of the organic acids. SPSS 25.0 software was used for significance analysis, SIMCA 14.1 software was used for PCA analysis and OPLS-DA analysis. For significance analysis, the normality of the data is evaluated first. Datasets conforming to normality assumptions proceeded to homogeneity of variance testing. Where variance homogeneity was satisfied, one-way analysis of variance (ANOVA) was performed, followed by the Least Significant Difference (LSD) post hoc test for pairwise comparisons. For normally distributed datasets exhibiting significant variance heterogeneity, Dunnett’s T3 test was employed for rigorous pairwise comparisons. Non-normally distributed datasets were analyzed using non-parametric alternatives such as the Kruskal–Wallis H test.

## 3. Results

### 3.1. Analysis of Non-Free Oxalic Acid

#### 3.1.1. Determination of Non-Free Oxalic Acid in Whole GG and MFCG with Different Growth Years

**Figure 1:**
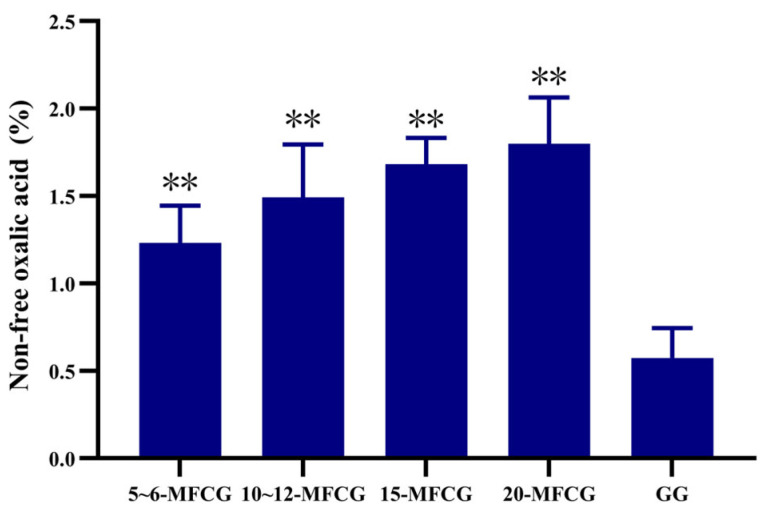
Average content of non-free oxalic acid in MFCG and GG. GG was significantly different as determined. ** *p* < 0.01.

Results indicated a significantly higher content of non-free oxalic acid in MFCG compared to GG, irrespective of growth years, and the results are shown in Figure 1. The content in MFCG was more than two times greater, with highly significant differences observed (*p* < 0.01). The content of non-free oxalic acid exhibited an increasing trend with longer growth years in MFCG. This discovery provides valuable insights into the dynamic nature of oxalic acid metabolism in ginseng.

#### 3.1.2. Determination of Non-Free Oxalic Acid in Different Parts of GG and MFCG with Different Growth Years

**Figure 2:**
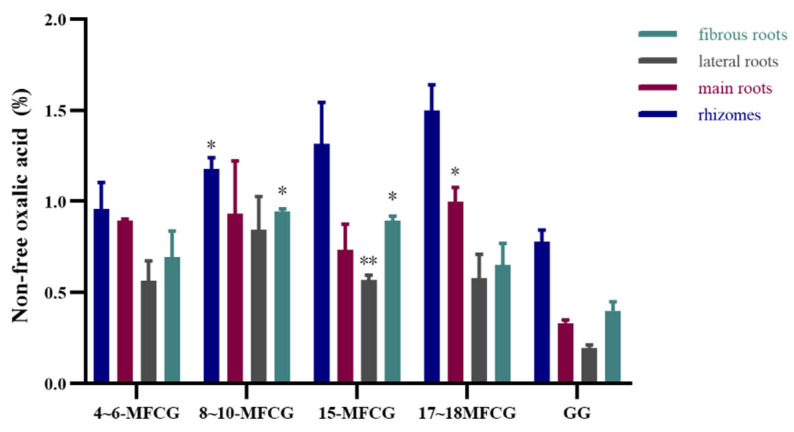
Average content of non-free oxalic acid in different parts of forest ginseng and garden ginseng with different growth years. GG was significantly different as determined. * *p* < 0.05, ** *p* < 0.01.

Results indicated that the distribution of non-free oxalic acid content in various parts of MFCG followed the order: rhizome > main root > fibrous root > lateral root, and the results are shown in Figure 2. Since MFCG has heavier rhizome than GG, the non-free oxalic acid content of MFCG and GG is different at different growth stages. Specifically, the content of non-free oxalic acid in all parts of 4–6-year-old MFCG was higher compared to that of 5-year-old GG, although these differences were not statistically significant. When compared to the content of non-free oxalic acid in the rhizome of GG, significantly higher content was observed in the rhizome of 8–10-year-old MFCG, with significant differences (*p* < 0.05). The content in the rhizome of 15-year-old and 17–18-year-old MFCG was even higher, although these differences were not statistically significant. Similarly, when compared to the content in the fibrous root of GG, the fibrous root of 8–10-year-old MFCG and 15-year-old MFCG displayed significantly higher content, with significant differences (*p* < 0.05).

Among different parts of ginseng, the content of non-free oxalic acid in rhizome was the highest, and it showed an increasing trend with the increase in age in MFCG. Therefore, when the growth years of MFCG increase, the weight of its rhizome increases, and its non-free oxalic acid content will also increase. In addition to that, the content in the main root remained relatively stable across different growth years. The lateral root demonstrated peak values of non-free oxalic acid content from 8 to 10 growth years, with minimal changes observed in other growth years. The fibrous root displayed an initial increasing trend in content from 4 to 10 growth years, followed by a subsequent decrease from 10 to 17 growth years.

### 3.2. Analysis of Calcium Oxalate Crystals

#### 3.2.1. Determination of Calcium Oxalate Crystals in Whole GG and MFCG with Different Growth Years

**Figure 3:**
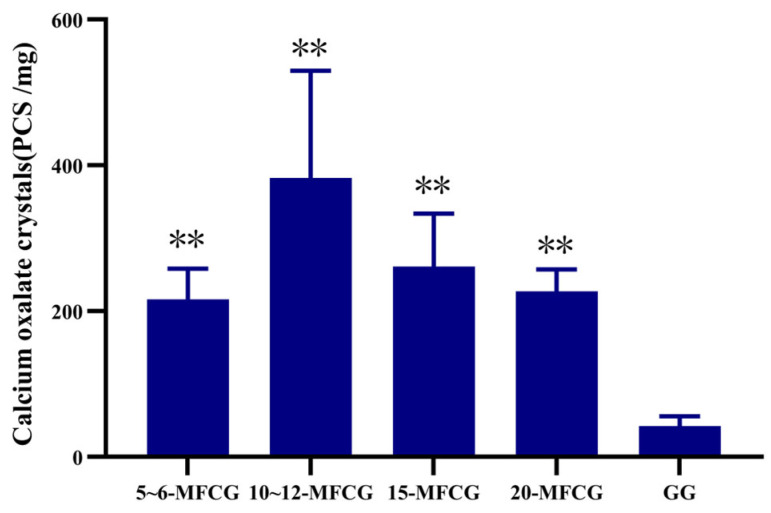
Average content of calcium oxalate crystals in MFCG and GG. GG was significantly different as determined. ** *p* < 0.01.

The results of the study revealed significant distinctions in oxalate crystal content between MFCG and GG, providing a novel basis for their differentiation, and the results are shown in Figure 3. Results indicated a significantly higher content of oxalate crystals in MFCG compared to GG, irrespective of growth years. Highly significant differences were observed (*p* < 0.01). The oxalate crystals content in MFCG varies with different growth years. The oxalate crystal content shows an upward trend in 5 to 10 years and a downward trend in 10 to 20 years.

#### 3.2.2. Determination of Calcium Oxalate Crystals in Different Parts of GG and MFCG with Different Growth Years

**Figure 4:**
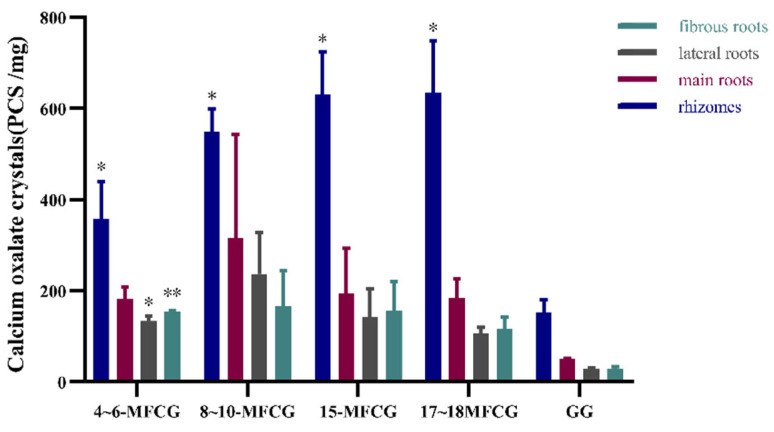
Average content of calcium oxalate crystals in different parts of GG and MFCG with different growth years. GG was significantly different as determined. * *p* < 0.05, ** *p* < 0.01.

The result of the study revealed intriguing insights into the distribution of calcium oxalate crystal content within different parts of MFCG, and the results are shown in Figure 4. The results show that the order of calcium oxalate crystal content in different parts is as follows: the root and stem, main root, lateral root, and fibrous root have the lowest content, and the contents are comparable. Due to the longer and heavier rhizome, the concentration of calcium oxalate crystals in MFCG is higher than that in GG. Compared with the content of calcium oxalate in GG, the content in MFCG samples of 4~6-year-old ginseng was significantly higher than that of GG (*p* < 0.05). The content of calcium oxalate in MFCG was higher in samples over 8 years old, and the difference was statistically significant (*p* < 0.05). These findings provide a marker for distinguishing the two ginseng varieties, while highlighting and emphasizing the uniqueness of MFCG.

The content of calcium oxalate crystals within the rhizome of MFCG exhibited an increasing trend as the growth years progressed. This suggests a potential correlation between crystal accumulation and the developmental stage of the rhizome. Conversely, the content in the main root remained relatively stable. The lateral root demonstrated peak values of calcium oxalate crystals content between 8 and 10 growth years, followed by a decreasing trend thereafter. Notably, the fibrous root demonstrated consistent content levels, suggesting a sustained metabolic equilibrium.

### 3.3. Analysis of Organic Acids

#### 3.3.1. Determination of Organic Acids in Whole GG and MFCG with Different Growth Years

**Figure 5:**
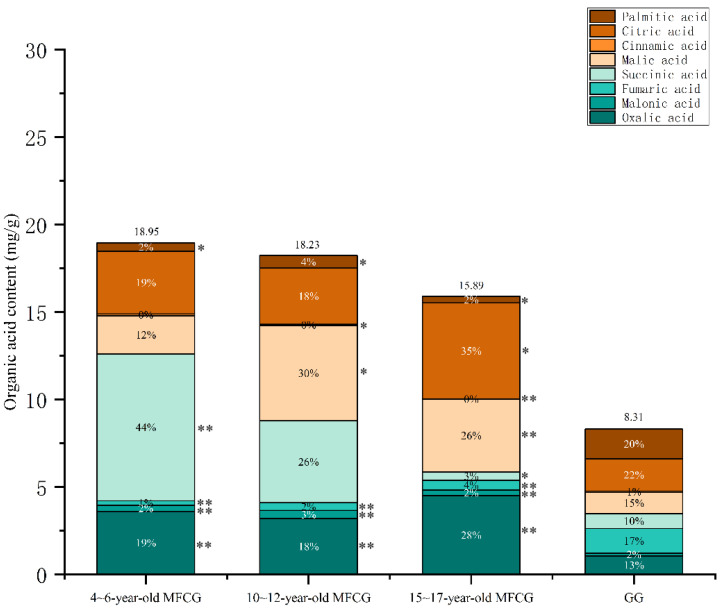
Average content of organic acids in MFCG and GG. GG was significantly different as determined. * *p* < 0.05, ** *p* < 0.01.

Statistical software SPSS 25 was used to test the significance of the difference in organic acids content between GG and MFCG in different growth years (Figure 5). The results showed that between 5-year-old MFCG and GG, there were significant differences in oxalic acid, malonic acid, fumaric acid, and succinic acid (*p* < 0.01), with significant differences in palmitic acid (*p* < 0.05). Between 10-year-old MFCG and GG, the contents of oxalic acid, malonic acid, and fumaric acid were significantly different (*p* < 0.01), and the malic acid, cinnamic acid, and palmitic acid were significantly different (*p* < 0.05). Between 15-year-old MFCG and GG, there were significant differences in the contents of oxalic acid, malonic acid, fumaric acid, malic acid, and cinnamic acid (*p* < 0.01), and succinic acid, citric acid, and palmitic acid were significantly different (*p* < 0.05). The fumaric acid content of MFCG in all growth years was lower than GG, while the total organic acids content was higher than GG. The fumaric acid ratio of total organic acids in MFCG was less than 9% and was greater than 9% in GG. The results of the study revealed significant distinctions in organic acids content between MFCG and GG, providing a novel basis for their differentiation.

#### 3.3.2. Determination of Organic Acids in Different Parts of GG and MFCG with Different Growth Years

**Figure 6:**
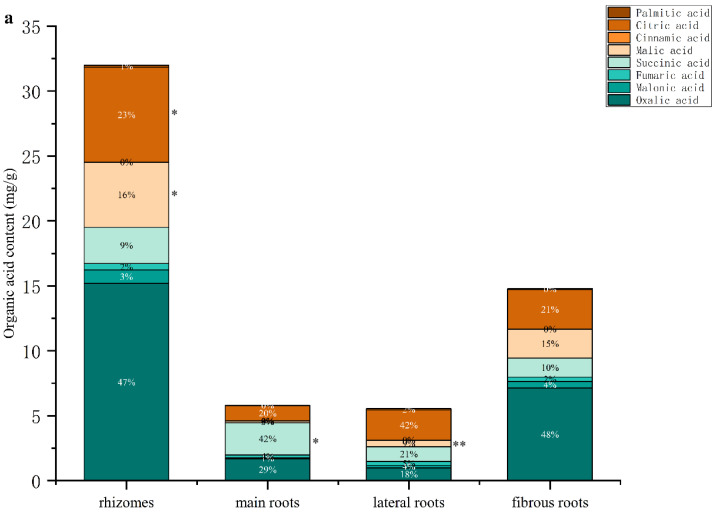

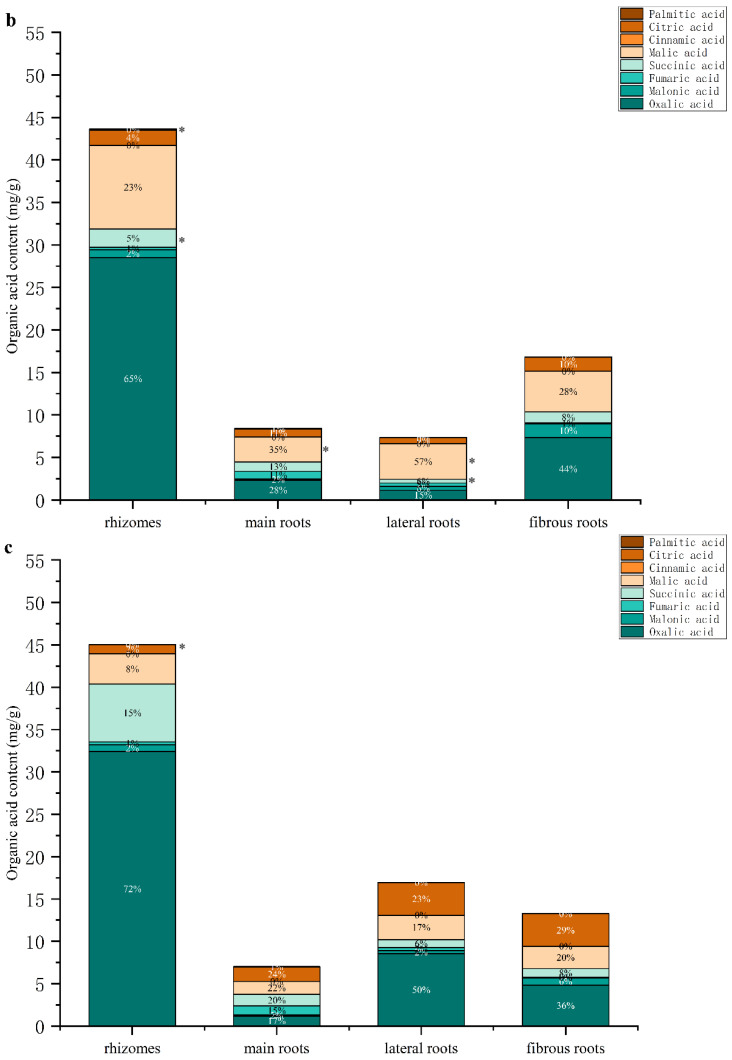

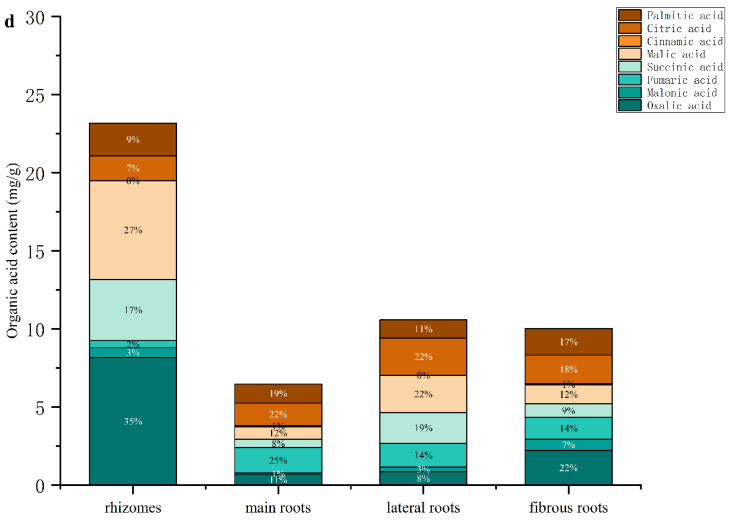
Average content of organic acids in different parts of GG and MFCG with different growth years. (**a**): 4~6-year-old MFCG; (**b**): 10~12-year-old MFCG; (**c**): 15~17-year-old MFCG; (**d**): GG. GG was significantly different as determined. * *p* < 0.05, ** *p* < 0.01.

Statistical software SPSS 25 was used to test the significance of the difference in organic acids content within different parts between GG and MFCG in different growth years (Figure 6). The contents of palmitic acid in rhizome of MFCG were significantly different from those of GG at any age (*p* < 0.05). The contents of malic acid and citric acid in the rhizome of 4~6-year-old MFCG were significantly different from those of GG (*p* < 0.05). The contents of succinic acid in the rhizome of 10~12-year-old MFCG were significantly different from those of GG (*p* < 0.05). The contents of succinic acid in the main root of 4~6-year-old MFCG were significantly different from those of GG (*p* < 0.05). The contents of malic acid in the main root of 10~12-year-old MFCG were significantly different from those of GG (*p* < 0.05). The contents of malic acid in the later root of 4~6-year-old MFCG were significantly different from those of GG (*p* < 0.01). The contents of succinic acid and malic acid in the later root of 10~12-year-old MFCG were significantly different from those of GG (*p* < 0.05).

### 3.4. Analysis of Ginsenosides

#### 3.4.1. Determination of Ginsenosides in Whole MFCG with Different Growth Years

**Figure 7:**
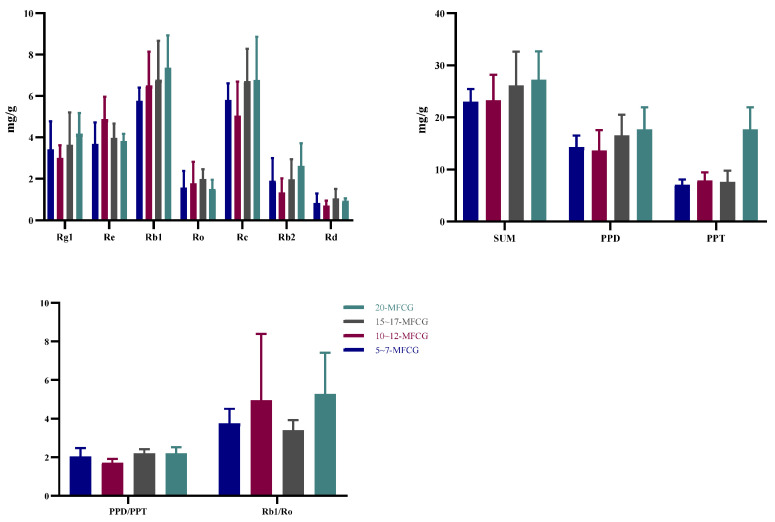
Ginsenosides in whole branches of MFCG with different growth years.

Analysis of the content and ratio of ginsenosides (Data are expressed as means ± standard errors) in MFCG with different growth years (Figure 7). From the above results, it can be seen that during 5~20 years of MFCG, the content of Rg1 shows a trend of first decreasing and then increasing with the increase in the growth period of MFCG, the content of Re shows a trend of first increasing and then decreasing with the increase in the growth period of MFCG, and the content of Rb1 shows a trend of first increasing, then decreasing and increasing again. In most cases, the total content of major ginsenosides is less than 25 mg/g for those under 10 years old, and ≥25 mg/g for those over 10 years old. The results show that the sum of the main ginsenosides is related to the growth years and can be used as a basis for preliminarily inferring the growth years of MFCG.

#### 3.4.2. Determination of Ginsenosides in Whole GG and MFCG

**Figure 8:**
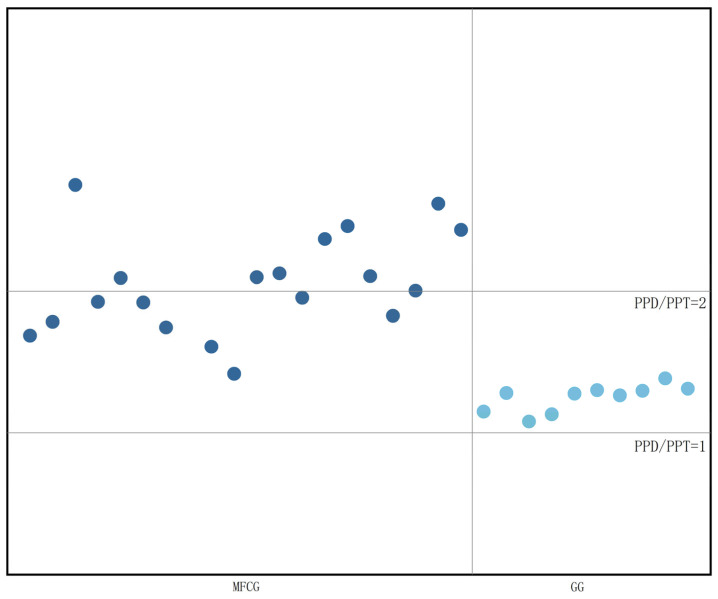
Ginsenoside ratio PPD/PPT of MFCG and GG.

**Figure 9:**
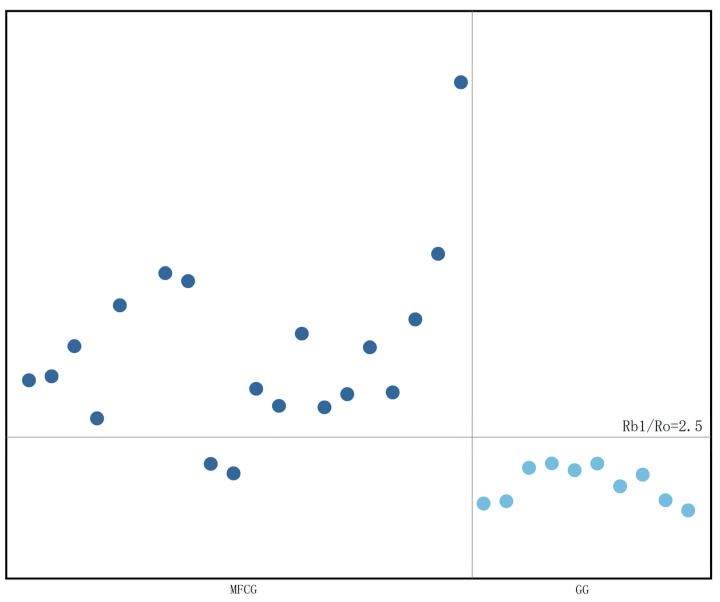
Ginsenoside ratio Rb1/Ro of MFCG and GG.

SPSS 20.0 was applied to test the significance of the difference in ginsenoside content between MFCG and GG (For each indicator *t*-test was used for normally distributed data of each index, and non-parametric test was used for non-normally distributed data). As can be seen from Table S10, there were highly significant differences between MFCG and GG in Rg1, Rb1, Rc, PPD, PPD/PPT, and Rb1/Ro (*p* < 0.01), there were significant differences in SUM between MFCG and GG (*p* < 0.05), there was no significant difference in Re, Ro, Rb2, Rd and PPT between MFCG and GG (*p* > 0.05). As can be seen from Figure 8 and Figure 9, in most cases, the content of Rb1 in MFCG is ≥6 mg/g, the ginsenoside ratio PPD/PPT is close to 2, and the ginsenoside ratio Rb1/Ro ≥ 2.5, while in GG, the content of Rb1 is <6 mg/g, the ginsenoside ratio PPD/PPT is close to 1, and the ginsenoside ratio Rb1/Ro < 2.5. These can be used as the basis for distinguishing between GG and MFCG.

#### 3.4.3. Determination of Ginsenosides in Different Parts of GG and MFCG

Observe the content and proportion of ginsenosides in MFCG and GG, and compare the differences in different parts: As can be seen from Table S12 (data expressed as mean ± standard error), the Rg1 and Ro of different parts of MFCG: rhizome > fibrous root > main root > lateral root; Re, Rb1, Rc, Rb2, Rd, SUM, PPD, PPT, PPD/PPT: fibrous root > rhizome > lateral root > main root; Rb1/Ro: fibrous root > lateral root > main root > rhizome. To sum up, among the different parts of MFCG, Rg1 and Ro are the highest in the rhizome, while the content and proportion of other ginsenosides are the highest in the fibrous root. This study systematically discussed the content and proportion of ginsenosides in different parts of MFCG.

As can be seen from Table S13 (data expressed as mean ± standard error), Rg1 in different parts of GG: rhizome > lateral root > main root > fibrous root; Re, Rb1, Rc, Rb2, SUM, PPD, PPT, PPD/PPT: fiber root > rhizome > lateral root > main root; Ro: Rhizome > fibrous root > lateral root > main root; Rd: Fibrous root > lateral root > rhizome > main root; Rb1/Ro: Fibrous root > lateral root > main root > rhizome. To sum up, among different parts of GG, Rg1 and Ro, which are similar to MFCG, have the highest content in the rhizome, while the content and proportion of other ginsenosides are the highest in the fibrous root.

Since both MFCG and GG have a larger proportion of the main root, and the difference in the appearance of the rhizome between MFCG and GG is more obvious, the proportion of ginsenosides Rb1/Ro and PPD/PPT in the total weight of MFCG is larger than that of GG. Therefore, the ratio of ginsenosides Rb1/Ro and PPD/PPT in the main root and rhizome is compared. The Rb1/Ro ratio in the rhizome and main root of MFCG is greater than that of the GG. Therefore, in the entire stem, the Rb1/Ro value of MFCG is greater than that of the GG, showing a pattern where the value of the MFCGt is greater than 2.5 and that of the GG is less than 2.5. The PPD/PPT ratio in the main root and rhizome of GG is close to 1, and that of MFCG is close to 2. Therefore, in the whole branch, it shows a pattern that the PPD/PPT ratio in GG is close to 1, and that in MFCG is close to 2.

### 3.5. Chemometric Analysis

#### 3.5.1. Non-Free Oxalic Acid and Calcium Oxalate Crystals Chemometric Analysis of MFCG and GG

**Figure 10:**
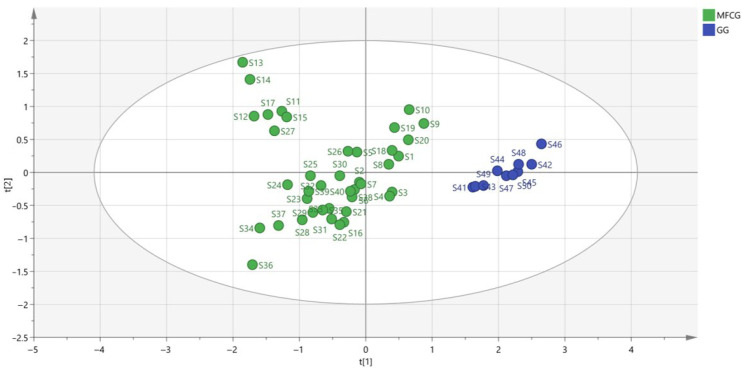
OPLS-DA score plot for oxalic acid of MFCG and GG.

To find novel marker substances to distinguish MFCG and GG, supervised OPLS-DA was performed using the data of MFCG and GG. In OPLS-DA, R2Y = 0.703 and Q2 = 0.693, indicating that the model is stable and reliable, and has a certain predictive ability. After the model was created, the data were displayed as OPLS-DA score plots (Figure 10). As shown, MFCG of different years and GG were very clearly clustered into two groups. Thus, to explore the potential markers that contributed most to the observed differences between the MFCG and GG, HPLC-DAD data were processed via supervised OPLS-DA with SIMCA software, and a VIP score was obtained (Table 2).

According to the variable importance in the project (VIP) >1 obtained by the OPLS-DA model, the ingredients with significant differences were screened out in Table 2. This suggests that non-free oxalic acid (%) significantly affected the categorization of MFCG and GG.

#### 3.5.2. Organic Acid Chemometric Analysis of MFCG and GG

**Figure 11:**
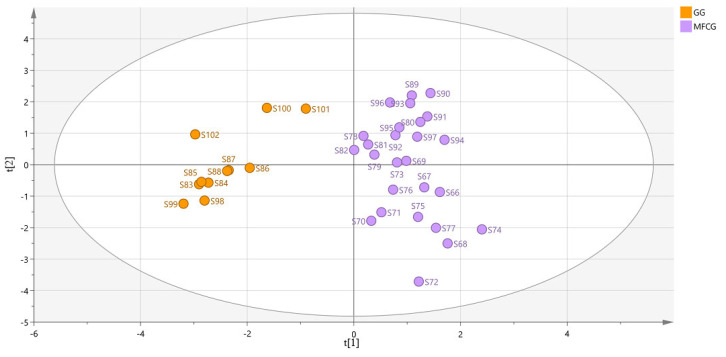
OPLS-DA score plot for organic acids of GG and MFCG.

To find novel marker substances of organic acids to distinguish MFCG and GG, supervised OPLS-DA was performed using the data of MFCG and GG. In OPLS-DA, R2Y = 0.88 and Q2 = 0.792, indicating that the model is stable and reliable, and has a certain predictive ability. After the model was created, the data were displayed as OPLS-DA score plots (Figure 11). As shown, MFCG of different years and GG were very clearly clustered into two groups. Thus, to explore the potential markers that contributed most to the observed differences between the MFCG and GG, HPLC-DAD data were processed via supervised OPLS-DA with SIMCA software, and a VIP score was obtained (Table 3).

According to the variable importance in the project (VIP) >1 obtained by the OPLS-DA model, the ingredients with significant differences were screened out in Table 3. This suggests that fumaric acid, malonic acid, total organic acid, and palmitic acid significantly affected the categorization of MFCG and GG, while the other factors have a relatively small effect. Therefore, fumaric acid/total organic acids (%) is used as the signature substance to distinguish between GG and MFCG.

#### 3.5.3. Organic Acid Chemometric Analysis of MFCG in Different Years

**Figure 12:**
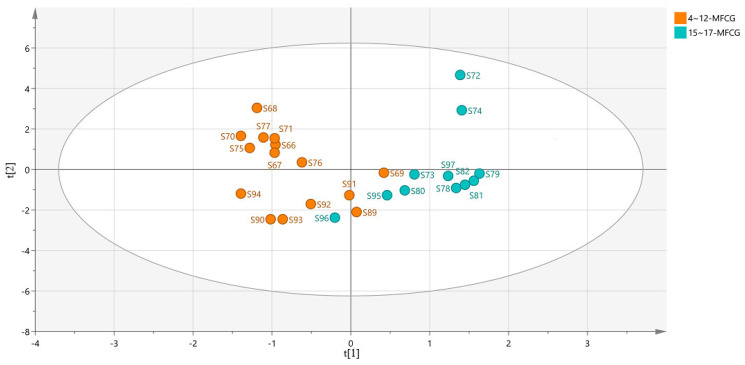
OPLS-DA score plot for organic acids of MFCG.

To find novel marker substances to distinguish MFCG of different years, supervised OPLS-DA was performed using the organic acids data of MFCG. In OPLS-DA, R2Y = 0.744 and Q2 = 0.622, indicating that the model is stable and reliable, and has a certain predictive ability. We divided MFCG into two groups, one with a growth age of 15~17 years, and the other with a growth age of 4~12 years. As shown, MFCG of different years were clustered into two groups (Figure 12). Thus, to explore the potential markers that contributed most to the observed differences between the MFCG of different years, GC/MS/MS data were processed via supervised OPLS-DA with SIMCA software, and a VIP score was obtained (Table 4).

According to the variable importance in the project (VIP) >1 obtained by the OPLS-DA model, the ingredients with significant differences were screened out in Table 4. This suggests that succinic acid, citric acid, total organic acids, and malonic acid play a role in distinguishing 15~17 years of MFCG from 4~12 years of MFCG, while the other factors have a relatively small effect.

#### 3.5.4. Ginsenosides Chemometric Analysis of MFCG and GG

**Figure 13:**
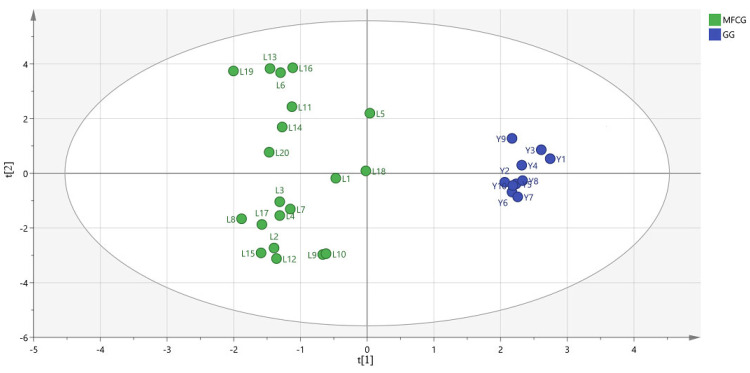
OPLS-DA score plot for ginsenosides of GG and MFCG.

To find novel marker substances to distinguish MFCG and GG, supervised OPLS-DA was performed using the ginsenosides data of MFCG and GG. In OPLS-DA, R2Y = 0.929 and Q2 = 0.889, indicating that the model is stable and reliable, and has a certain predictive ability. We divided MFCG and GG into two groups (Figure 13). Thus, to explore the potential markers that contributed most to the observed differences between the MFCG and GG, ginsenosides data were processed via supervised OPLS-DA with SIMCA software, and a VIP score was obtained (Table 5).

According to the variable importance in the project (VIP) >1 obtained by the OPLS-DA model, the ingredients with significant differences were screened out in Table 5. This suggests that PPD/PPT, Rb1, Rb1/Ro, and PPD play a role in distinguishing MFCG from GG, while the other factors have a relatively small effect.

### 3.6. Correlation Analysis of Pharmacognosy Data and Each Component

#### 3.6.1. Correlation Analysis of Pharmacognosy Data with Non-Free Oxalic Acid and Calcium Oxalate Crystal

Spearman correlation analysis was conducted on the appearance trait indicators of MFCG and GG (rhizome length and weight; main root length and weight, lateral root length and weight, fibrous root weight and total weight, Table S14) and intrinsic quality indicators (non-free oxalic acid and calcium oxalate crystal content). As can be seen from Table S15, in MFCG, non-free oxalic acid was positively correlated with the growing age, the length of rhizome, the weight of rhizome, and the length of main root (ρ = 0.641 **, 0.661 **, 0.421 **, 0.411 **, *p* < 0.01). Non-free oxalic acid was positively correlated with the weight of main root (ρ = 0.340 *, *p* < 0.05). Non-free oxalic acid was negatively correlated with the length of lateral root (ρ = −0.405 **, *p* < 0.01). Calcium oxalate crystal was positively correlated with the weight of rhizome (ρ = 0.369 *, *p* < 0.05). As can be seen from Table S16, in GG, Non-free oxalic acid was positively correlated with the weight of fibrous root (ρ = 0.673 *, *p* < 0.05).

#### 3.6.2. Correlation Analysis of Pharmacognosy Data and Organic Acids

Spearman correlation analysis was conducted on the appearance trait indicators of MFCG and GG (rhizome length and weight; main root length and weight, lateral root length and weight, fibrous root weight and total weight, Table S14) and intrinsic quality indicators (organic acids content and proportion). As can be seen from Table S17, in MFCG, fumaric acid was positively correlated with the weight of lateral root (ρ = 0.482 *, *p* < 0.05). Succinic acid was negatively correlated with the growth year (ρ = −0.649 **, *p* < 0.01), negatively correlated with the length of rhizome (ρ = −0.467 *, *p* < 0.05), and positively correlated with the length of the lateral root (ρ = 0.409 *, *p* < 0.05). Cinnamic acid was negatively correlated with the growth year (ρ = −0.418 *, *p* < 0.05). Citric acid was negatively correlated with the length of lateral root (ρ = −0.482 *, *p* < 0.05). Fumaric acid/total organic acid was positively correlated with the weight of lateral root (ρ = 0.426 *, *p* < 0.05).

As can be seen from Table S18, in GG, fumaric acid was negatively correlated with the length of rhizome and the weight of fibrous root (ρ = −0.816 *, −0.609 *, *p* < 0.05). Cinnamic acid was positively correlated with the weight of rhizome (ρ = 0.615 *, *p* < 0.05). Palmitic acid was positively correlated with the weight of rhizome (ρ = 0.773 **, *p* < 0.01). Palmitic acid was positively correlated with the length of rhizome and the weight of lateral root (ρ = 0.645 *, 0.681 *, *p* < 0.05). Fumaric acid/total organic acid was negatively correlated with the length of rhizome and the weight of fibrous root (ρ = −0.807 **, −0.745 **, *p* < 0.01). Fumaric acid/total organic acid was negatively correlated with the weight of rhizome (ρ = −0.700 *, *p* < 0.05).

#### 3.6.3. Correlation Analysis of Pharmacognosy Data and Ginsenosides

Spearman correlation analysis was conducted on the appearance trait indicators of MFCG and GG (rhizome length and weight; main root length and weight, lateral root length and weight, fibrous root weight and total weight, Table S14) and intrinsic quality indicators (ginsenoside content and proportion). As can be seen from Table S19, in MFCG, the content of ginsenoside Rg1 was positively correlated with the weight of lateral root (ρ = 0.456 *, *p* < 0.05). The ginsenoside Re content was negatively correlated with the weight of the rhizome and the total weight (ρ = −0.447 *, −0.541 *, *p* < 0.05). The ginsenoside Ro content was positively correlated with the length of lateral root (ρ = 0.549 *, *p* < 0.05). The content of ginsenoside Rd was positively correlated with the weight of fibrous root (ρ = 0.475 *, *p* < 0.05). The content of PPD was positively correlated with the weight of lateral root (ρ = 0.483 *, *p* < 0.05). PPD/PPT was positively correlated with the weight of the root head, the weight of the lateral root, the weight of the fibrous root, and the total weight (ρ = 0.595 **, 0.630 **, 0.564 **, 0.648 **, *p* < 0.01).

As can be seen from Table S20, in GG, the content of ginsenoside Ro was positively correlated with the weight of lateral root (ρ = 0.721 *, *p* < 0.05). The ginsenoside Rc content was positively correlated with the weight of main root and growth year (ρ = 0.661 *, 0.696 *, *p* < 0.05). The ginsenoside Rb2 content was negatively correlated with the weight of main root, growth year, and total weight (ρ = −0.770 **, −0.696 *, −0.697 *, *p* < 0.05). The ginsenoside PPD content was positively correlated with the weight of main root, growth year, and total weight (ρ = 0.758 *, 0.696 *, 0.648 *, *p* < 0.05). PPD/PPT was positively correlated with the growth year (ρ = 0.696 **, *p* < 0.05).

## 4. Discussion

Oxalic acid is of great significance in the field of chemistry [17,18,19]. Oxalic acid has the functions of regulating growth and development, enhancing defense responses, and detoxifying heavy metals under biological and abiotic stresses [10,13,20]. Meanwhile, oxalic acid has been proven to affect the content of other chemical components within plants [11,21]. The length of the rhizome of ginseng can reflect its age. The rhizome of MFCG is longer than that of GG. Therefore, the changes in the length and weight of the rhizome may explain the changes in oxalate content. This discovery indicates a potential connection between the age of ginseng and oxalic acid metabolism. This is because the withering and regeneration of the rhizome during the growth of ginseng can also lead to the formation of scars, which in turn causes an increase in the scale of the rhizome and is conducive to the accumulation of calcium oxalate crystals and non-free oxalic acid. Studies have shown that in plants such as Norwegian spruce and desert lily, the formation and accumulation of calcium oxalate crystals are part of their defense mechanisms [14,22]. To counter the damage from nature such as herbivores, these plants generate and accumulate large amounts of calcium oxalate crystals in the already injured areas, thereby preventing further attacks from animals. Similarly, the withering and regrowth of the rhizome during the growth of ginseng contributes to the increase in rhizome scales, which is conducive to the accumulation of calcium oxalate crystals.

Organic acids are a class of compounds produced during the growth and metabolism of plants, playing a significant role in their growth and development, physiological regulation, and adaptation to the environment. Among them, malonic acid is a regulator of metabolism and signal transduction. Succinic acid is an intermediate in the tricarboxylic acid cycle and has antioxidant properties. Fumaric acid is also an intermediate in the tricarboxylic acid cycle, participating in energy metabolism and regulating acid-base balance. Citric acid is one of the most abundant organic acids in ginseng and has the functions of regulating pH value and promoting the transportation of nutrients. Palmitic acid is a saturated fatty acid that can help plants store energy and maintain the structure of plant cell membranes [23]. These organic acids play a certain role in the growth and development of plants and their adaptation to environmental stress. The experimental results of the GC-MS/MS study indicate that fumaric acid/total organic acid (%) is the characteristic component ratio between MFCG and GG. The results indicated that compared with MFCG, the content ratio of fumaric acid to total organic acid (%) in GG was significantly higher than that in MFCG, and was independent of the growth year, suggesting that this was the key content ratio for distinguishing the two types of ginseng. The contents of organic acids in different growth years and different parts of the MFCG of GG vary, revealing the distribution of these organic acids in ginseng.

The content of ginsenosides is closely related to factors such as the growth environment, growth cycle, and artificial intervention, and it is closely related to the quality of ginseng [24]. During the years covered by this study, the contents of ginsenosides basically showed a gradually increasing trend with the growth years. The total content of ginsenosides in MFCG is higher than that in GG, which is consistent with the results reported by previous researchers [25]. Studies show that the higher the content of ginsenosides, the greater their medicinal and economic value. There is a significant quality difference between MFCG and GG, and MFCG is more notable in terms of medicinal and economic value. The content of ginsenosides varies in different parts of ginseng. The rhizomes of MFCG are longer and of greater quality, while those of GG have more fibrous roots. In the determination of ginsenoside content in different parts of ginseng, it was found that ginsenoside Ro had the highest content in the rhizome of the MFCG and GG, which was consistent with the reports [26]. Ginsenoside Re has the highest content in the fibrous roots of MFCG and GG, which is consistent with the research results [27]. In this study, it was found that the content of ginsenoside Rb1, the ratio of ginsenoside Rb1/Ro, and PPD/PPT could distinguish MFCG and GG. MFCG grows naturally in wild environments such as mountains and forests. The growth conditions of MFCG are relatively poor, while those of GG are relatively better. Under environmental stress, MFCG is more adversely affected. Due to adverse effects and environmental influences, MFCG accumulated more ginsenosides, and the ginsenoside ratio was different from that of GG.

This study took ginsenosides, the active component in ginseng, and organic acids, the characteristic component in ginseng, as the research objects. By determining their contents and combining chemometrics analysis, MFCG and GG were distinguished and identified. Compared with the previous methods, it can ensure accuracy while using simpler instruments, a quicker method and strong universality. In the stoichiometric analysis of oxalic acid, non-free oxalic acid has a significant influence in distinguishing MFCG from GG, and this result is consistent with our de-termination data. In the stoichiometric analysis of GC-MS/MS, fumaric acid, malonic acid, palmitic acid, and total organic acids have significant influences on the classification of MFCG and GG. Meanwhile, the contents of succinic acid, citric acid, and malonic acid are closely related to the growth years of MFCG. In the stoichiometric analysis of ginsenosides, PPD/PPT, Rb1, Rb1/Ro, and PPD play a role in distinguishing MFCG from GG. These results can provide certain support for our further conclusion.

## 5. Conclusions

The research results reveal the differences in oxalate, organic acid, and ginsenosides contents between MFCG and GG, providing a new basis for their identification. It is recommended that the content of non-free oxalate in MFCG should be higher than 0.8%, the content of GG should be kept below this threshold. Comparative analysis revealed that the content of non-free oxalic acid was the highest in the rhizome of MFCG. It is recommended that the content of calcium oxalate in MFCG should not be less than 160/mg, while the aggregated content of calcium oxalate in GG should not exceed 60/mg. The analysis of calcium oxalate crystals from different parts of ginseng revealed that the content was the highest in the rhizome, followed by the main root, and the lowest in the lateral root and fibrous root. The proposed standard dictates that the content of fumaric acid/total organic acids in GG should ideally fall at or above 9%, whereas that of MFCG should maintain levels below this threshold. Succinic acid, citric acid, and malonic acid play a role in distinguishing 15~17 years of MFCG from 4~12 years of MFCG, which can provide a reference for the differentiation of MFCG at different ages. In most cases, the content of Rb1 in MFCG is ≥6 mg/g, the ginsenoside ratio PPD/PPT is close to 2, and the ginsenoside ratio Rb1/Ro ≥ 2.5, while in GG, the content of Rb1 is <6 mg/g, the ginsenoside ratio PPD/PPT is close to 1, and the ginsenoside ratio Rb1/Ro < 2.5. In the analysis of ginsenoside content in different parts of MFCG and GG, Rg1 and Ro have the highest content in the rhizome, while the content and proportion of other ginsenosides are the highest in the fibrous root. The ginseng samples used in this study were from different origins, and the content determination results of the ginseng samples from each origin all met the thresholds proposed in this study. It is indicated that the differences between MFCG and GG are only influenced by growth period and growth environment, and the geographic origin and cultivation practices do not overly affect their component contents. Although the sample sources selected for this study were different, the determination of the relevant contents of samples from other origins or growth environments can still be further improved. To the best of our knowledge, this is the first study to report the disparities in non-free oxalate acid, calcium oxalate crystals, organic acids, and ginsenoside between MFCG and GG. As far as we know, the present study is the first to determine the difference in the ratio of ginsenosides (Rb1/Ro, PPD/PPT) and the ratio of organic acids, which can accurately and rapidly identify MFCG from GG, providing a basic framework for the quality control plans of MFCG and laying a scientific foundation for formulating effective quality control measures for MFCG.

## Figures and Tables

**Table 1:** Retention time and ion pairs of each organic acid.

Organic Acids	Methyl Ester Derivative	Retention Time (min)	Ion Pair	Collision Energy
Oxalic acid	Dimethyl oxalate	8.42	118.1/59.0, 59.1/43.0	5, 5
Malonic acid	Dimethyl malonate	11.993	101.1/59.0, 101.1/41.8	5, 25
Fumaric acid	Dimethyl fumarate	15.654	113.1/85.0, 113.1/53.0	5, 15
Succinic acid	Dimethyl succinate	16.074	115.1/87.1, 115.1/55.0	5, 15
Malic acid	Dimethyl malate	19.626	103.1/70.9, 103.1/61.0	5, 5
Adipic acid	Dimethyl adipate	23.834	114.1/71.0, 114.1/43.0	15, 15
Cinnamic acid	Methyl cinnamate	28.541	131.1/103.1, 131.1/77.0	5, 25
Citric acid	Trimethyl citrate	31.171	143.1/101.0, 143.1/59.0	5, 15
Palmitic acid	Methyl palmitate	40.986	74.1/43.1, 227.1/73.0	5, 5

**Table 2:** VIP value of OPLS-DA of each ingredient in oxalic acid and calcium oxalate crystals.

Ingredient	VIP Value
Non-free oxalic acid (%)	1.01507
Calcium oxalate crystals (PCS/mg)	0.98470

**Table 3:** VIP value of OPLS-DA of each ingredient in organic acids of GG and MFCG.

Ingredient	VIP Value
Fumaric acid	1.24443
Malonic acid	1.13807
Total organic acid	1.09166
Palmitic acid	1.08190
Malic acid	0.96832
Citric acid	0.91866
Oxalic acid	0.87792
Succinic acid	0.86379
Cinnamic acid	0.70392

**Table 4:** VIP value of OPLS-DA of each ingredient in organic acids of MFCG.

Ingredient	VIP Value
Succinic acid	1.50191
Citric acid	1.2061
Malonic acid	1.03052
Cinnamic acid	0.988088
Fumaric acid	0.950071
Palmitic acid	0.899554
Total organic acid	0.89132
Malic acid	0.688544
Oxalic acid	0.520494

**Table 5:** VIP value of OPLS-DA of each ingredient in ginsenosides.

Ingredient	VIP Value
PPD/PPT	1.38342
Rb1	1.36364
Rb1/Ro	1.14347
PPD	1.14306
Rb2	0.930154
Rg1	0.921191
SUM	0.917947
PPT	0.862362
Ro	0.799999
Rc	0.772205
Rd	0.750548
Re	0.71612

## Data Availability

The original contributions presented in the study are included in the article and supplementary material, further inquiries can be directed to the corresponding author.

## Supplementary Materials

The following supporting information can be downloaded at: https://www.mdpi.com/article/10.3390/foods14173073/s1, Table S1: Details of the GG and MFCG samples; Table S2: Standard curve and the recovery of oxalic acid; Table S3: Standard curve and the recovery of organic acid; Table S4: Standard curve and the recovery of ginsenosides; Table S5: Contents of non-free oxalic acid and calcium oxalate crystals in the whole ginseng (MFCG and GG); Table S6: Contents of non-free oxalic acid and calcium oxalate crystals in different parts of ginseng (MFCG and GG); Table S7: Contents of organic acid in the whole ginseng (MFCG and GG); Table S8: Contents of organic acid in different parts of ginseng (MFCG and GG); Table S9: Contents of ginsenosides in the whole ginseng (MFCG and GG); Table S10: Significant analysis of the differences in ginsenosides between MFCG and GG; Table S11: Contents of ginsenosides in different parts of ginseng (MFCG and GG); Table S12: Determination of ginsenosides in different parts of MFCG; Table S13: Determination of ginsenosides in different parts of GG; Table S14: Pharmacognostic data of ginseng appearance; Table S15: Correlation analysis of pharmacognosy data with non-free oxalic acid and calcium oxalate crystal in MFCG; Table S16: Correlation analysis of pharmacognosy data with non-free oxalic acid and calcium oxalate crystal in GG; Table S17: Correlation Analysis Results between the appearance traits of MFCG and organic acid content; Table S18: Correlation Analysis Results between the appearance traits of GG and organic acid content; Table S19: Correlation Analysis Results between the appearance traits of MFCG and ginsenoside content; Table S20: Correlation Analysis Results between the appearance traits of GG and ginsenoside content; Figure S1: Chromatogram of oxalic acid in the sample; Figure S2: Chromatogram of organic acids in the sample.; Figure S3: Chromatogram of ginsenosides in the sample; Figure S4 Sample solutions preparation for HPLC determination of non-free oxalic acid; Figure S5 Sample solutions preparation for microscopic identification; Figure S6 Sample solutions preparation for GC-MS/MS; Figure S7 Sample solutions preparation for HPLC determination of ginsenosides.
