# Supplementary material for: The Distinction of Chemical Profiles of Mountainous Forest Cultivated Ginseng and Garden Ginseng Based on Calcium Oxalate Crystals, Organic Acids, and Ginsenosides
Source: Foods. 2025 Aug 30;14(17):3073. doi: 10.3390/foods14173073 (PMC12427741; doi:10.3390/foods14173073)
Supplement: Supplementary file 1 [file foods-14-03073-s001.zip › foods-3834608-supplementary.pdf]

Supplementary material

1 Details of the GG and MFCG samples

Table S1

Details of the GG and MFCG samples

Kind	Sample No.	Growth years	Source
MFCG	S1~S8	5	HR
MFCG	S9~S10	6	JA
MFCG	S11~S17	10	HR
MFCG	S18~S19	10	JA
MFCG	S20	12	JA
MFCG	S21~S30	15	HR
MFCG	S31~S40	20	HR
GG	S41~S50	5	JL
MFCG	S51~S53	4~6	XB
MFCG	S54~S56	8~10	XB
MFCG	S57~S59	15	XB
MFCG	S60~S62	17~18	HR
GG	S63~S65	5	JL
MFCG	S66	5	HR
MFCG	S67~S68	4~6	BS
MFCG	S69	10	HR
MFCG	S70~S71	10~12	BS
MFCG	S72~S73	15	KD
MFCG	S74	17	JA
MFCG	S75~S77	11~12	HR
MFCG	S78~S82	15	JA
GG	S83~S88	5	BS
MFCG	S89~S91	5	KD
MFCG	S92~S94	10	KD
MFCG	S95~97	15	KD
GG	S98~99	5	BS
GG	S100~102	5	HR
MFCG	S103~S105	5~7	HR
MFCG	S106~S108	10~12	HR
MFCG	S109~S111	15~17	HR
MFCG	S112~S114	⩾20	HR
GG	S115~S120	5	JL
GG	S121	9	KD
MFCG	S122~S123	5~7	HR
MFCG	S124~S125	10~12	HR
MFCG	S126~S127	15~17	HR

MFCG	S128~S129	≥20	HR
GG	S130~SS131	5	JL
GG	S132	9	KD

Note: HR- Huanren Manchu Autonomous County, Liaoning Province, China; JA-JiAn City, Jilin Province, China; JL- Jilin Province, China; XB-Xinbin Manchu Autonomous County, Liaoning Province, China; KD-Kuandian Manchu Autonomous County, Liaoning Province, China; BS-BaiShan City, Jilin Province, China

2 Validation of the method on the determination of oxalic acid

An excellent linear correlation existed between the peak area and concentration of the standard (r>0.9996) in the tested concentration range. Results showed that the precision, repeatability, and stability of the instrument were good and met the requirements. For the precision test, the RSD of the six samples were all below 0.518%. The repeatability presented as RSD (n=6) was below 0.692%. For the stability test, the RSD of the six samples was below 0.702%. The recoveries of the oxalic acid ranged between 98.9～103.5%. (Table S2).

Table S2

Standard curve and the recovery of oxalic acid

	Standard curve	Average recovery(%)	RSD(%)
oxalic acid	Y=639.6X-15.212	101.80	1.899

3 Validation of the method on the determination of organic acid

The determination was carried out according to the above-mentioned chromatographic conditions. An excellent linear correlation existed between the peak area and concentration of each standard in the tested concentration range. Results showed that the precision, repeatability, and stability of the instrument were good and met the requirements. For the precision test, the RSD of the six samples were all below 1.609%. The repeatability presented as RSD(n=6) was below 1.962%. For the stability test, the RSD of the six samples was below 1.915%. The recoveries of the organic acids ranged between 95～105%. (Table S3).

Table S3

Standard curve and the recovery of organic acid

	Standard curve	R2	Linear range	Average recovery(%)	RSD(%)
Oxalic acid	Y=3.385X-3.736	0.9993	0.024~4.80	101.26	1.966
Malonic acid	Y=33.90X-1.090	0.9991	0.002~3.40	101.53	2.156

Fumaric acid	Y=3.950X-0.552	0.9992	0.019~0.75	100.59	2.579
Succinic acid	Y=0.550X-0.542	0.9993	0.030~5.92	99.79	1.897
Malic acid	Y=22.02X-16.13	0.9992	0.163~3.26	100.63	2.430
Cinnamic acid	Y=9.750X-0.552	0.9985	0.007~1.30	99.77	1.384
Citric acid	Y=48.00X-9.949	0.9993	0.017~3.45	100.83	2.585
Palmitic acid	Y=7.454X-0.919	0.9994	0.003~0.57	97.67	1.421

4 Validation of the method on the determination of ginsenosides

An excellent linear correlation existed between the peak area and concentration of the standard (r>0.9993) in the tested concentration range. Results showed that the precision, repeatability, and stability of the instrument were good and met the requirements. For the precision test, the RSD of the six samples were all below 1.580%. The repeatability presented as RSD (n=6) was below 1.529%. For the stability test, the RSD of the six samples was below 1.442%. The recoveries of the ginsenosides ranged between 100.55～101.58%. (Table S4).

Table S4

Standard curve and the recovery of ginsenosides

	Standard curve	R2	Linear range	Average recovery(%)	RSD(%)
Rg1	y=2947.4x+1.1968	0.9999	0.500～16.000	101.58	0.460
Re	y=2223.1x+3.911	0.9997	0.493～15.760	101.49	1.200
Rb1	y=2062.3x+10.48	0.9999	0.500～16.000	101.54	1.104
Rc	y=2609.3x+7.5633	0.9993	0.515～16.480	100.91	1.154
Ro	y=3960.4x+12.782	0.9999	0.505～16.160	100.66	0.917
Rb2	y=3266.9x+45.92	0.9994	0.510～16.320	100.55	1.002
Rd	y=3814.6x+11.021	0.9997	0518～16.560	100.96	0.972

5 Analysis of non-free oxalic acid and calcium oxalate crystals

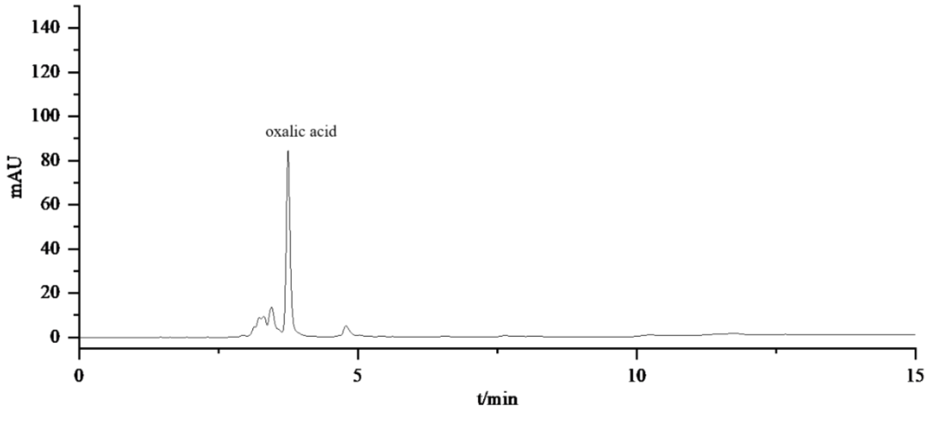

Figure S1 Chromatogram of oxalic acid in the sample

Table S5

Contents of non-free oxalic acid and calcium oxalate crystals in the whole ginseng (MFCG and GG)

NO.	Kind	Non-free oxalic acid (%)	Calcium oxalate crystals content (PCS /mg)
S1	MFCG	1.12	211.13
S2	MFCG	1.42	216.64
S3	MFCG	1.28	156.63
S4	MFCG	1.30	151.83
S5	MFCG	1.34	274.71
S6	MFCG	1.48	209.46
S7	MFCG	1.42	211.66
S8	MFCG	1.20	209.96
S9	MFCG	0.87	237.14
S10	MFCG	0.90	281.32
S11	MFCG	1.62	445.75
S12	MFCG	1.79	473.38
S13	MFCG	1.67	584.84
S14	MFCG	1.69	544.20
S15	MFCG	1.61	428.73
S16	MFCG	1.65	165.18
S17	MFCG	1.71	457.54
S18	MFCG	1.14	231.29
S19	MFCG	1.04	268.06
S20	MFCG	1.01	227.92
S21	MFCG	1.60	180.93
S22	MFCG	1.68	167.07
S23	MFCG	1.77	255.59
S24	MFCG	1.83	305.84
S25	MFCG	1.67	292.75
S26	MFCG	1.38	287.81
S27	MFCG	1.72	419.96
S28	MFCG	1.87	224.80
S29	MFCG	1.79	223.23
S30	MFCG	1.52	254.26
S31	MFCG	1.71	187.14
S32	MFCG	1.65	261.80
S33	MFCG	1.68	209.19
S34	MFCG	2.14	265.65
S35	MFCG	1.72	215.48
S36	MFCG	2.30	208.86
S37	MFCG	2.02	245.39
S38	MFCG	1.52	200.94
S39	MFCG	1.74	267.27
S40	MFCG	1.50	213.06
S41	GG	0.81	59.17

S42	GG	0.40	22.11
S43	GG	0.79	57.21
S44	GG	0.61	56.62
S45	GG	0.50	27.64
S46	GG	0.28	46.41
S47	GG	0.58	36.12
S48	GG	0.48	40.54
S49	GG	0.74	47.64
S50	GG	0.54	28.21

Table S6

Contents of non-free oxalic acid and calcium oxalate crystals in different parts of ginseng (MFCG and GG)

NO.	Kind	Non-free oxalic acid (%)	Calcium oxalate crystals content (PCS /mg)
S51a	MFCG	1.01	328.32
S51b	MFCG	0.90	184.81
S51c	MFCG	0.48	134.71
S51d	MFCG	0.84	150.61
S52a	MFCG	0.79	297.38
S52b	MFCG	0.89	153.73
S52c	MFCG	0.69	143.95
S52d	MFCG	0.69	156.09
S53a	MFCG	1.07	449.59
S53b	MFCG	0.90	205.61
S53c	MFCG	0.52	122.40
S53d	MFCG	0.56	155.47
S54a	MFCG	1.11	582.87
S54b	MFCG	0.60	242.88
S54c	MFCG	0.78	253.44
S54d	MFCG	0.96	254.80
S55a	MFCG	1.22	490.10
S55b	MFCG	1.03	134.93
S55c	MFCG	0.70	139.45
S55d	MFCG	0.93	137.78
S56a	MFCG	1.21	572.63
S56b	MFCG	1.16	570.33
S56c	MFCG	1.05	319.04
S56d	MFCG	0.94	105.98
S57a	MFCG	1.11	548.67
S57b	MFCG	0.83	188.13
S57c	MFCG	0.59	78.14
S57d	MFCG	0.87	224.05
S58a	MFCG	1.56	614.12
S58b	MFCG	0.57	98.29

S58c	MFCG	0.54	153.35
S58d	MFCG	0.89	149.09
S59a	MFCG	1.28	730.88
S59b	MFCG	0.80	295.69
S59c	MFCG	0.58	197.64
S59d	MFCG	0.92	96.75
S60a	MFCG	1.52	559.95
S60b	MFCG	0.91	223.55
S60c	MFCG	0.73	121.48
S60d	MFCG	0.65	130.88
S61a	MFCG	1.35	577.51
S61b	MFCG	1.01	189.47
S61c	MFCG	0.50	94.79
S61d	MFCG	0.77	130.64
S62a	MFCG	1.63	765.04
S62b	MFCG	1.07	138.89
S62c	MFCG	0.51	102.49
S62d	MFCG	0.54	86.68
S63a	GG	0.76	135.52
S63b	GG	0.35	51.45
S63c	GG	0.21	28.48
S63d	GG	0.45	34.52
S64a	GG	0.85	184.53
S64b	GG	0.32	49.75
S64c	GG	0.18	29.85
S64d	GG	0.35	26.20
S65a	GG	0.72	136.65
S65b	GG	0.33	50.19
S65c	GG	0.20	25.86
S65d	GG	0.40	25.92

Note: a-rhizome, b-main root, c-lateral root, d-fibrous root.

6 Analysis of organic acid

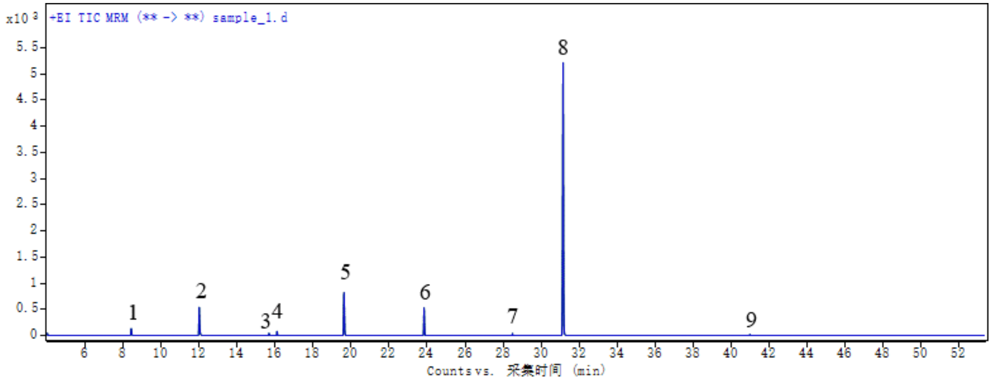

Figure S2 Chromatogram of organic acids in the sample. 1-oxalic acid; 2-malonic acid; 3-fumaric acid; 4-succinic acid; 5-malic acid; 6-adipic acid; 7-cinnamic acid; 8-citric acid; 9- palmitic acid

Table S7

Contents of organic acid in the whole ginseng (MFCG and GG)

NO.	Kind	Oxalic acid content (mg/g)	Malonic acid content (mg/g)	Fumaric acid content (mg/g)	Succinic acid content (mg/g)	Malic acid content (mg/g)	Cinnamic acid content (mg/g)	Citric acid content (mg/g)	Palmitic acid content (mg/g)	Total organic acid content (mg/g)	Fumaric acid/Total organic acid content (%)
S66	MFCG	3.908	0.358	0.271	13.618	3.191	0.226	3.986	0.719	26.277	1.031
S67	MFCG	3.141	0.366	0.162	15.726	2.801	0.000	3.963	0.625	26.784	0.605
S68	MFCG	3.015	0.488	0.200	15.189	4.103	0.338	6.580	1.274	31.187	0.641
S69	MFCG	0.947	0.414	0.449	0.459	3.824	0.000	6.853	0.348	13.293	3.379
S70	MFCG	4.125	0.483	0.150	13.427	2.806	0.000	3.083	1.883	25.958	0.579

S71	MFCG	3.012	0.409	1.049	8.510	2.653	0.647	3.033	1.481	20.794	5.045
S72	MFCG	5.726	0.618	0.000	0.000	7.853	0.039	12.166	1.894	28.295	0.000
S73	MFCG	3.584	0.328	0.604	0.030	6.406	0.000	4.693	0.234	15.879	3.801
S74	MFCG	5.865	0.500	0.000	0.000	9.829	0.000	10.719	0.357	27.271	0.000
S75	MFCG	1.709	0.535	0.204	6.331	9.097	0.000	4.362	0.858	23.095	0.884
S76	MFCG	1.854	0.357	0.262	5.350	7.355	0.000	3.434	0.864	19.476	1.346
S77	MFCG	2.508	0.551	0.079	5.007	10.749	0.000	5.115	0.844	24.854	0.319
S78	MFCG	3.739	0.233	0.767	0.073	2.413	0.000	4.252	0.366	11.843	6.475
S79	MFCG	4.129	0.277	0.775	0.000	3.687	0.000	5.570	0.317	14.754	5.252
S80	MFCG	1.545	0.237	0.221	0.000	1.892	0.000	5.989	0.124	10.008	2.211
S81	MFCG	2.738	0.262	0.866	0.092	3.645	0.000	5.111	0.136	12.850	6.735
S82	MFCG	3.962	0.249	0.902	0.233	3.567	0.000	4.413	0.381	13.707	6.580
S83	GG	1.490	0.183	1.660	1.107	1.276	0.126	2.023	1.989	9.854	16.845
S84	GG	1.261	0.148	1.509	0.914	1.253	0.149	2.317	2.048	9.598	15.721
S85	GG	1.344	0.164	1.518	0.664	1.400	0.084	2.125	2.033	9.333	16.262
S86	GG	1.050	0.140	0.908	0.729	1.171	0.081	2.086	2.077	8.242	11.018
S87	GG	0.913	0.169	1.045	0.781	1.094	0.033	1.719	2.156	7.910	13.214
S88	GG	0.913	0.162	1.065	0.998	0.959	0.034	1.817	2.144	8.093	13.161
S89	MFCG	2.918	0.155	0.251	2.088	1.129	0.002	2.347	0.058	8.949	2.806
S90	MFCG	1.820	0.313	0.186	1.747	1.030	0.004	2.000	0.016	7.116	2.619
S91	MFCG	6.846	0.394	0.449	2.045	0.885	0.000	2.676	0.121	13.416	3.349
S92	MFCG	2.537	0.341	0.631	1.232	6.270	0.000	1.294	0.045	12.350	5.112
S93	MFCG	4.036	0.374	0.519	0.666	2.615	0.000	0.489	0.028	8.728	5.947
S94	MFCG	8.082	0.711	0.624	1.147	3.637	0.000	1.251	0.111	15.563	4.010
S95	MFCG	1.284	0.202	0.441	2.348	3.399	0.000	4.115	0.054	11.843	3.724

S96	MFCG	3.274	0.312	0.666	0.732	1.624	0.000	1.421	0.027	8.056	8.271
S97	MFCG	13.696	0.459	0.895	1.536	1.710	0.000	2.064	0.090	20.451	4.378
S98	GG	0.808	0.143	1.017	1.192	1.141	0.188	1.701	3.003	9.194	11.488
S99	GG	0.862	0.158	1.186	1.173	1.270	0.130	1.573	2.996	9.348	13.486
S100	GG	0.839	0.076	1.674	0.566	1.612	0.000	1.031	0.074	5.873	34.691
S101	GG	0.826	0.085	1.342	0.805	1.053	0.021	2.650	0.023	6.803	21.657
S102	GG	1.337	0.238	2.641	0.579	1.127	0.000	1.167	0.127	7.216	43.301

Note: S89~S102 is the whole content of different parts.

Table S8

Contents of organic acid in different parts of ginseng (MFCG and GG)

NO.	Kind	Oxalic acid content (mg/g)	Malonic acid content (mg/g)	Fumaric acid content (mg/g)	Succinic acid content (mg/g)	Malic acid content (mg/g)	Cinnamic acid content (mg/g)	Citric acid content (mg/g)	Palmitic acid content (mg/g)	Total organic acid content (mg/g)	Fumaric acid/Total organic acid content (%)
S89a	MFCG	1.598	0.764	0.427	3.589	7.665	0.000	12.556	0.218	26.817	1.592
S89b	MFCG	1.425	0.065	0.194	2.423	0.207	0.004	1.087	0.021	5.426	3.577
S89c	MFCG	1.313	0.131	0.236	1.401	0.663	0.000	2.713	0.099	6.555	3.601
S89d	MFCG	12.396	0.224	0.407	1.242	2.082	0.000	1.122	0.038	17.510	2.322
S90a	MFCG	5.244	1.592	0.077	1.925	2.700	0.000	5.102	0.052	16.690	0.459
S90b	MFCG	0.504	0.002	0.173	2.359	0.155	0.000	1.063	0.005	4.261	4.069
S90c	MFCG	0.479	0.193	0.220	0.993	0.586	0.000	1.730	0.001	4.201	5.228
S90d	MFCG	5.417	0.148	0.268	1.499	2.998	0.028	2.116	0.050	12.524	2.140
S91a	MFCG	38.705	0.778	1.007	2.899	4.669	0.000	4.237	0.247	52.542	1.916

S91b	MFCG	3.104	0.140	0.361	2.601	0.092	0.000	1.407	0.036	7.742	4.659
S91c	MFCG	1.190	0.277	0.414	1.039	0.248	0.000	2.528	0.193	5.888	7.029
S91d	MFCG	3.565	1.173	0.330	1.612	1.603	0.000	5.912	0.136	14.332	2.302
S92a	MFCG	16.796	0.695	0.316	2.414	13.522	0.000	2.177	0.105	36.024	0.876
S92b	MFCG	1.241	0.136	1.015	1.283	4.491	0.000	1.166	0.062	9.395	10.808
S92c	MFCG	0.083	0.333	0.348	0.686	6.538	0.000	0.959	0.007	8.955	3.883
S92d	MFCG	2.771	0.918	0.111	1.579	6.713	0.000	2.039	0.029	14.161	0.787
S93a	MFCG	22.813	0.624	0.245	1.681	7.795	0.000	0.993	0.036	34.186	0.716
S93b	MFCG	2.057	0.088	0.816	0.814	1.644	0.000	0.572	0.036	6.027	13.546
S93c	MFCG	0.689	0.354	0.294	0.105	2.564	0.000	0.023	0.010	4.039	7.280
S93d	MFCG	7.338	1.562	0.046	0.806	2.869	0.000	1.084	0.042	13.748	0.334
S94a	MFCG	45.963	1.379	0.334	2.362	8.192	0.000	2.275	0.176	60.681	0.550
S94b	MFCG	3.660	0.222	0.832	1.269	2.614	0.000	1.051	0.136	9.784	8.506
S94c	MFCG	2.617	0.718	0.532	0.498	3.517	0.000	1.074	0.055	9.010	5.901
S94d	MFCG	11.908	2.317	0.222	1.542	4.658	0.000	1.808	0.113	22.568	0.982
S95a	MFCG	1.368	0.164	0.842	3.540	5.002	0.000	1.670	0.061	12.649	6.661
S95b	MFCG	1.996	0.262	0.792	3.647	3.567	0.000	0.685	0.067	11.017	7.191
S95c	MFCG	0.473	0.132	0.005	0.865	2.882	0.000	7.502	0.038	11.897	0.041
S95d	MFCG	0.644	0.175	0.000	0.565	3.117	0.000	9.748	0.043	14.291	0.000
S96a	MFCG	47.651	0.966	0.000	9.094	2.740	0.000	0.609	0.040	61.100	0.000
S96b	MFCG	0.496	0.021	1.361	0.191	0.499	0.000	1.330	0.044	3.943	34.520
S96c	MFCG	0.000	0.391	0.000	0.000	2.742	0.000	1.882	0.000	5.015	0.000
S96d	MFCG	0.150	1.016	0.000	0.603	2.558	0.000	0.834	0.024	5.186	0.000
S97a	MFCG	48.255	1.290	0.068	7.973	2.867	0.000	0.920	0.041	61.414	0.111
S97b	MFCG	1.058	0.060	1.080	0.286	0.558	0.000	2.979	0.095	6.118	17.659

S97c	MFCG	25.186	0.577	1.032	1.942	2.993	0.000	1.346	0.096	33.172	3.111
S97d	MFCG	13.730	1.350	0.178	1.909	2.206	0.000	0.900	0.084	20.356	0.873
S98a	GG	1.242	0.468	0.089	3.309	2.730	0.002	2.561	4.139	14.541	0.615
S98b	GG	0.632	0.083	0.981	0.904	0.688	0.239	1.535	3.043	8.106	12.106
S98c	GG	0.800	0.260	1.421	1.781	2.222	0.054	2.130	2.369	11.035	12.873
S98d	GG	3.614	0.370	0.820	1.497	2.792	0.067	1.863	3.962	14.986	5.474
S99a	GG	1.120	0.464	0.085	3.174	2.772	0.000	2.594	3.599	13.808	0.613
S99b	GG	0.758	0.103	1.244	0.883	0.748	0.171	1.180	2.856	7.943	15.657
S99c	GG	0.718	0.244	1.232	1.706	2.321	0.041	2.562	3.279	12.103	10.181
S99d	GG	3.599	0.376	0.805	1.314	2.930	0.065	1.605	3.256	13.950	5.770
S100a	GG	6.444	0.512	0.708	2.702	11.210	0.000	0.920	0.832	23.330	3.037
S100b	GG	0.594	0.016	1.735	0.257	1.111	0.000	0.687	0.051	4.452	38.983
S100c	GG	1.171	0.288	1.755	2.067	3.016	0.000	3.134	0.075	11.505	15.250
S100d	GG	2.219	0.762	0.352	2.644	3.408	0.000	3.909	0.123	13.418	2.626
S101a	GG	14.957	0.549	0.077	7.450	5.728	0.000	0.828	0.013	29.602	0.259
S101b	GG	0.511	0.045	1.505	0.345	0.696	0.025	3.030	0.023	6.179	24.352
S101c	GG	0.054	0.197	0.630	2.428	2.373	0.000	0.621	0.023	6.326	9.961
S101d	GG	1.191	0.538	0.111	2.718	2.808	0.000	0.474	0.031	7.871	1.412
S102a	GG	17.109	1.103	1.363	3.009	9.142	0.000	0.992	1.850	34.568	3.943
S102b	GG	0.882	0.142	2.744	0.278	0.782	0.000	0.781	0.075	5.683	48.294
S102c	GG	1.520	0.528	2.558	1.912	1.922	0.000	3.396	0.121	11.957	21.393
S102d	GG	0.500	1.504	0.594	2.761	1.591	0.000	5.018	0.276	12.243	4.848

Note: a-rhizome, b-main root, c-lateral root, d-fibrous root.

7 Analysis of ginsenosides

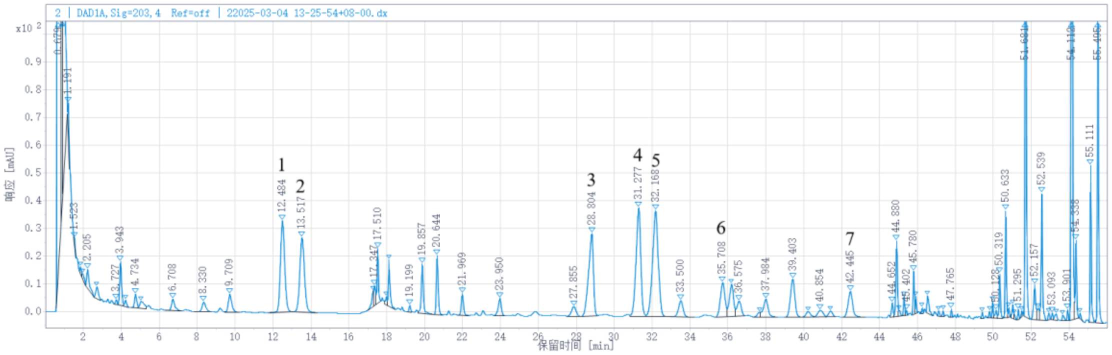

Figure S3 Chromatogram of ginsenosides in the sample.1-Rg1、2-Re、3- Rb1、4-Ro、5-Rc、6-Rb2、7-Rd

Table S9

Contents of ginsenosides in the whole ginseng (MFCG and GG)

NO.	Kind	Rg1	Re	Rb1	Ro	Rc	Rb2	Rd	SUM	PPD	PPT	PPD/PPT	Rb1/Ro
S103	MFCG	2.944	5.129	5.400	1.543	6.148	0.810	1.257	23.231	13.615	8.073	1.686	3.499
S104	MFCG	2.480	4.191	4.932	1.382	5.479	1.396	0.088	19.947	11.894	6.671	1.783	3.570
S105	MFCG	2.133	3.743	5.780	1.410	6.390	3.195	0.802	23.454	16.167	5.877	2.751	4.098
S122	MFCG	4.033	2.657	6.192	2.191	4.563	1.135	0.990	21.761	12.880	6.690	1.925	2.827
S123	MFCG	5.486	2.670	6.543	1.359	6.482	2.975	1.068	26.583	17.068	8.156	2.093	4.816
S106	MFCG	3.939	6.707	9.298	0.902	7.648	2.527	0.964	31.985	20.436	10.646	1.920	10.303
S107	MFCG	3.355	4.013	5.588	1.038	5.523	1.298	0.432	21.247	12.841	7.368	1.743	5.384
S108	MFCG	2.659	4.372	6.520	1.243	4.565	1.031	0.958	21.349	13.074	7.031	1.860	5.244
S124	MFCG	2.465	4.314	5.176	2.553	4.114	0.965	0.645	20.232	10.901	6.779	1.608	2.028
S125	MFCG	2.585	5.043	5.989	3.220	3.385	0.886	0.546	21.653	10.805	7.628	1.416	1.860

S109	MFCG	4.340	4.558	7.241	2.162	8.505	1.481	1.451	29.738	18.678	8.898	2.099	3.349
S110	MFCG	1.975	3.454	4.443	1.458	5.299	0.932	0.868	18.429	11.542	5.429	2.126	3.047
S111	MFCG	5.767	4.316	8.981	2.079	7.370	1.727	1.622	31.861	19.699	10.083	1.954	4.320
S126	MFCG	3.850	4.517	7.989	2.643	7.581	3.493	0.755	30.829	19.818	8.368	2.368	3.023
S127	MFCG	2.253	3.036	5.303	1.629	4.861	2.286	0.562	19.928	13.011	5.288	2.460	3.256
S112	MFCG	5.760	4.043	7.777	1.906	9.630	2.396	0.847	32.358	20.649	9.803	2.106	4.080
S113	MFCG	3.250	3.676	6.122	1.864	4.665	1.055	0.804	21.435	12.645	6.926	1.826	3.285
S114	MFCG	3.548	3.748	5.557	1.216	5.481	2.642	0.939	23.132	14.621	7.295	2.004	4.570
S128	MFCG	4.522	4.289	9.532	1.665	8.289	4.139	1.113	33.549	23.073	8.811	2.619	5.724
S129	MFCG	3.840	3.346	7.811	0.894	5.782	2.881	1.006	25.560	17.480	7.186	2.433	8.742
S115	GG	4.820	4.638	3.571	2.682	5.325	0.965	1.010	23.009	10.870	9.458	1.149	1.332
S116	GG	4.284	4.261	3.679	2.687	5.366	0.958	0.943	22.179	10.947	8.545	1.281	1.369
S117	GG	4.894	4.714	3.112	1.590	5.314	0.959	0.985	21.567	10.369	9.608	1.079	1.957
S118	GG	4.545	4.506	3.065	1.506	5.207	1.017	0.940	20.786	10.229	9.051	1.130	2.035
S119	GG	3.837	4.190	3.028	1.580	4.714	1.382	1.117	19.849	10.241	8.027	1.276	1.916
S120	GG	3.648	4.084	2.826	1.390	4.684	1.534	1.017	19.182	10.060	7.731	1.301	2.033
S130	GG	4.630	2.931	2.960	1.812	4.244	1.442	0.907	18.926	9.554	7.560	1.264	1.634
S131	GG	4.813	3.024	3.106	1.690	4.507	1.522	1.019	19.680	10.154	7.836	1.296	1.838
S121	GG	5.107	4.290	3.615	2.605	7.614	0.898	0.871	25.000	12.998	9.396	1.383	1.388
S132	GG	3.888	4.487	3.304	2.734	5.887	0.790	1.001	22.093	10.983	8.376	1.311	1.208

Note: S122~S132 is the whole content of different parts. SUM- the sum of the total ginsenosides contents, PPD-protopanaxadiol, PPT-protopanaxatriol.

Table S10

Significant analysis of the differences in ginsenosides between MFCG and GG

	GG	MFCG
Rg1	4.43±0.47	3.56±1.18**
Re	4.04±0.69	4.09±0.92
Rb1	3.17±0.30	6.61±1.50**
Ro	1.87±0.52	1.72±0.61
Rc	4.92±0.44	6.09±1.64**
Rb2	1.22±0.27	1.96±1.01
Rd	0.99±0.07	0.89±0.35
SUM	20.65±1.49	24.91±4.97*
PPD	10.30±0.45	15.54±3.78**
PPT	8.48±0.81	7.65±1.47
PPD/PPT	1.22±0.09	2.04±0.35**
Rb1/Ro	1.76±0.29	4.35±2.06**

Table S11

Contents of ginsenosides in different parts of ginseng (MFCG and GG)

NO.	Kind	Rg1	Re	Rb1	Ro	Rc	Rb2	Rd	SUM	PPD	PPT	PPD/PPT	Rb1/Ro
S122a	MFCG	8.251	7.915	15.138	10.466	14.920	4.159	2.377	63.224	36.593	16.166	2.264	1.446
S122b	MFCG	2.953	1.410	2.892	1.106	1.746	0.386	0.242	10.736	5.266	4.363	1.207	2.614
S122c	MFCG	4.165	2.479	6.576	1.853	4.544	0.873	1.118	21.607	13.111	6.644	1.973	3.550
S122d	MFCG	8.028	8.843	23.493	4.047	18.066	5.994	5.462	73.933	53.015	16.871	3.142	5.806
S123a	MFCG	6.971	5.824	13.325	6.194	16.247	5.821	1.526	55.908	36.920	12.795	2.885	2.151
S123b	MFCG	4.860	1.320	4.015	1.004	3.566	1.411	0.383	16.560	9.376	6.181	1.517	4.001
S123c	MFCG	6.645	2.921	7.089	0.956	8.042	3.319	1.125	30.097	19.575	9.566	2.046	7.418
S123d	MFCG	4.291	8.814	17.010	1.383	13.319	10.565	5.424	60.806	46.318	13.105	3.534	12.302

S124a	MFCG	4.105	8.077	8.379	11.131	9.316	2.786	1.025	44.819	21.506	12.182	1.765	0.753
S124b	MFCG	2.636	3.019	3.214	1.738	2.401	0.571	0.241	13.819	6.426	5.655	1.136	1.849
S124c	MFCG	1.776	2.829	3.309	1.360	2.272	0.474	0.324	12.344	6.379	4.605	1.385	2.433
S124d	MFCG	3.840	11.821	16.861	3.254	13.281	2.866	2.986	54.910	35.995	15.661	2.298	5.182
S125a	MFCG	4.194	9.557	9.081	12.763	7.977	1.559	0.827	45.959	19.445	13.751	1.414	0.712
S125b	MFCG	3.989	4.182	5.368	2.773	2.797	0.482	0.298	19.890	8.946	8.171	1.095	1.936
S125c	MFCG	1.391	3.395	3.609	1.456	1.540	0.808	0.278	12.476	6.235	4.785	1.303	2.480
S125d	MFCG	2.751	11.475	17.111	3.420	9.919	1.810	2.437	48.925	31.278	14.227	2.199	5.003
S126a	MFCG	4.916	6.783	10.991	7.965	11.229	4.144	0.870	46.898	27.235	11.699	2.328	1.380
S126b	MFCG	4.169	3.832	6.979	2.448	6.398	2.941	0.428	27.194	16.745	8.001	2.093	2.851
S126c	MFCG	3.602	3.024	5.641	1.861	5.065	2.404	0.364	21.961	13.473	6.626	2.033	3.031
S126d	MFCG	3.038	12.739	21.193	2.495	21.692	10.608	3.885	75.651	57.379	15.777	3.637	8.494
S127a	MFCG	4.169	5.849	9.043	5.921	9.176	3.743	0.718	38.619	22.680	10.018	2.264	1.527
S127b	MFCG	1.791	1.272	2.220	1.064	1.760	0.847	0.108	9.061	4.935	3.063	1.611	2.087
S127c	MFCG	2.161	2.611	5.245	1.045	4.669	2.253	0.433	18.418	12.600	4.773	2.640	5.018
S127d	MFCG	3.162	11.850	20.069	1.888	19.466	9.527	3.529	69.490	52.590	15.012	3.503	10.631
S128a	MFCG	5.148	4.081	10.113	2.653	8.527	3.948	0.892	35.361	23.479	9.229	2.544	3.811
S128b	MFCG	4.219	2.395	5.907	1.582	4.287	1.713	0.254	20.357	12.161	6.614	1.839	3.734
S128c	MFCG	3.540	2.448	5.885	0.767	4.500	2.161	0.284	19.585	12.830	5.988	2.142	7.673
S128d	MFCG	6.237	12.162	22.631	2.046	23.008	12.923	4.969	83.977	63.532	18.399	3.453	11.060
S129a	MFCG	5.687	4.083	10.392	1.850	7.460	3.511	0.994	33.977	22.357	9.770	2.288	5.617
S129b	MFCG	2.069	1.291	2.603	0.481	1.686	0.745	0.168	9.043	5.202	3.360	1.548	5.407
S129c	MFCG	2.344	1.476	3.400	0.221	2.299	1.429	0.276	11.446	7.404	3.820	1.938	15.377
S129d	MFCG	4.944	9.108	19.448	0.471	16.002	7.959	4.182	62.115	47.592	14.052	3.387	41.288
S130a	GG	5.274	5.983	6.027	5.486	8.373	2.825	1.619	35.586	18.844	11.257	1.674	1.099

S130b	GG	4.612	1.973	2.302	1.467	3.037	0.886	0.508	14.785	6.732	6.585	1.022	1.569
S130c	GG	4.851	4.702	3.966	2.251	7.067	2.376	1.632	26.844	15.041	9.553	1.575	1.762
S130d	GG	3.249	9.118	7.148	2.192	9.031	5.902	4.146	40.786	26.227	12.367	2.121	3.260
S131a	GG	5.690	6.546	6.007	4.691	8.387	2.467	1.747	35.535	18.608	12.237	1.521	1.281
S131b	GG	4.775	1.891	2.396	1.396	3.183	0.933	0.549	15.122	7.061	6.666	1.059	1.716
S131c	GG	4.962	4.779	4.080	2.100	7.103	2.391	1.746	27.161	15.320	9.741	1.573	1.943
S131d	GG	3.701	9.930	7.609	1.778	9.151	6.268	4.595	43.031	27.622	13.631	2.026	4.279
S132a	GG	4.003	5.649	3.075	7.990	7.945	1.236	0.685	30.583	12.941	9.652	1.341	0.385
S132b	GG	3.942	1.478	1.793	1.576	2.534	0.416	0.255	11.994	4.997	5.421	0.922	1.138
S132c	GG	4.107	4.373	2.851	2.733	5.748	0.753	0.797	21.362	10.149	8.479	1.197	1.043
S132d	GG	2.685	12.806	9.626	3.898	14.919	1.814	4.111	49.858	30.470	15.491	1.967	2.470

Note: a-rhizome, b-main root, c-lateral root, d-fibrous root. SUM- the sum of the total ginsenosides contents, PPD-protopanaxadiol, PPT-protopanaxatriol.

Table S12

Determination of ginsenosides in different parts of MFCG

	Rhizome	Main root	Lateral root	Fibrous root
Rg1	5.43±1.49	3.34±1.12	3.20±1.70	4.54±1.82
Re	6.52±1.95	2.34±1.21	2.65±0.57	10.85±1.64
Rb1	10.81±2.32	4.15±1.74	5.09±1.48	19.73±2.60
Ro	7.37±3.95	1.52±0.78	1.19±0.56	2.38±1.17
Rc	10.61±3.29	3.08±1.63	4.12±2.08	16.84±4.53
Rb2	3.71±1.22	1.14±0.86	1.72±0.98	7.78±3.93
Rd	1.15±0.55	0.27±0.10	0.53±0.37	4.11±1.12
SUM	45.60±10.06	15.83±6.42	18.49±6.34	66.23±11.64

PPD	26.28±6.83	8.63±4.15	11.45±4.56	48.46±10.69
PPT	11.95±2.32	5.68±1.95	5.85±1.81	15.39±1.69
PPD/PPT	2.22±0.45	1.51±0.35	1.93±0.43	3.14±0.57
Rb1/Ro	2.17±1.70	3.06±1.24	5.87±4.37	12.47±11.98

Table S13

Determination of ginsenosides in different parts of GG

	Rhizome	Main root	Lateral root	Fibrous root
Rg1	4.99±0.88	4.44±0.44	4.64±0.47	3.21±0.51
Re	6.06±0.45	1.78±0.27	4.62±0.22	10.62±1.94
Rb1	5.04±1.70	2.16±0.32	3.63±0.68	8.13±1.32
Ro	6.06±1.72	1.48±0.09	2.36±0.33	2.62±1.12
Rc	8.23±0.25	2.92±0.34	6.64±0.77	11.03±3.37
Rb2	2.18±0.83	0.74±0.29	1.84±0.94	4.66±2.47
Rd	1.35±0.58	0.44±0.16	1.39±0.52	4.28±0.27
SUM	33.90±2.87	13.97±1.72	25.12±3.26	44.56±4.73
PPD	16.80±3.34	6.26±1.11	13.50±2.91	28.11±2.16
PPT	11.05±1.30	6.22±0.70	9.26±0.68	13.83±1.57
PPD/PPT	1.51±0.17	1.00±0.07	1.45±0.22	2.04±0.08
Rb1/Ro	0.92±0.47	1.47±0.30	1.58±0.48	3.34±0.91

8 Pharmacognostic data of ginseng appearance

Table S14

Pharmacognostic data of ginseng appearance

NO.	Rhizome length (cm)	Rhizome weight (g)	Main root length (cm)	Main root weight (g)	Lateral root length (cm)	Lateral root weight (g)	Fibrous weight (g)	Total weight (g)
S1	1.4	0.0368	2.7	0.4453	7.0	0.1051	0.0090	0.5962
S2	2.3	0.0962	5.9	0.8883	4.1	0.1066	0.0070	1.0981
S3	1.5	0.0745	5.1	0.5302	2.5	0.0285	0.0358	0.6690
S4	2.2	0.1069	3.6	0.9950	6.6	0.1122	0.0755	1.2896
S5	2.8	0.1280	7.5	1.1346	9.5	0.0807	0.0327	1.3760
S6	2.5	0.0718	8.0	0.6629	4.7	0.0188	0.0289	0.7824
S7	1.3	0.0393	2.9	0.2674	3.6	0.1076	0.0061	0.4204
S8	2.0	0.0526	4.0	0.3697	2.5	0.0166	0.0000	0.4389
S9	1.8	0.2785	3.1	1.6026	3.7	1.9913	0.3709	4.2433
S10	3.2	0.3543	1.6	1.9270	8.8	4.3244	0.6178	7.2235
S11	3.8	0.2301	9.0	0.6289	3.5	0.0101	0.0217	0.8908
S12	5.2	0.5738	3.7	1.5530	4.3	0.3365	0.1061	2.5694
S13	3.0	0.2460	11.2	1.0550	5.0	0.0645	0.0578	1.4233
S14	2.4	0.4590	7.8	1.9950	4.0	0.1016	0.2751	2.8352
S15	2.5	0.4961	11.2	1.4584	2.9	0.2670	0.0809	2.3024
S16	2.2	0.2986	6.8	2.4096	6.5	0.3246	0.1229	3.1557
S17	3.0	0.2816	5.5	1.0467	1.5	0.2318	0.1309	1.6910
S18	2.6	0.4157	1.7	3.0539	6.0	2.2294	0.5713	6.2703
S19	2.4	0.6628	2.6	3.0529	2.5	1.6479	0.7155	6.0791
S20	3.1	0.2712	2.6	1.1806	4.4	1.8017	0.4113	3.6648
S21	3.9	0.5979	12.5	4.3486	5.1	0.6246	0.2416	5.8127
S22	3.6	0.4446	5.5	2.1183	3.9	0.5075	0.1566	3.2270
S23	4.3	0.7839	8.4	2.4467	6.5	0.5069	0.8808	4.6183

S24	4.0	0.6913	7.2	4.2398	0.0	0.0000	0.3751	5.3062
S25	3.5	0.5928	4.2	2.6528	3.7	0.5765	0.5266	4.3487
S26	2.1	0.6446	4.9	2.6255	0.0	0.0000	0.5525	3.8226
S27	4.3	0.4728	13.5	2.9331	0.0	0.0000	0.2575	3.6634
S28	2.9	0.2955	2.5	1.1808	2.0	1.1110	0.3334	2.9207
S29	3.2	0.2720	4.1	2.8201	6.2	0.9340	0.1379	4.1640
S30	2.1	0.1495	2.5	1.5578	2.4	0.0910	0.2217	2.0200
S31	2.4	0.1487	9.5	2.1284	0.0	0.0000	0.0726	2.3497
S32	4.7	0.6166	2.5	0.7787	6.5	0.7411	0.9644	3.1010
S33	4.0	0.1977	4.3	1.6585	3.7	0.1217	0.5418	2.1597
S34	6.2	0.4097	4.9	2.2118	0.0	0.0000	0.0410	2.6625
S35	3.1	0.4712	10.5	3.2755	0.0	0.0000	0.3081	4.0548
S36	7.6	0.8121	5.4	1.8054	2.9	0.4127	0.1214	3.1516
S37	5.9	0.3318	8.9	1.7863	0.0	0.0000	0.0463	2.1644
S38	3.1	0.4286	8.0	2.1553	4.5	0.1187	0.1653	2.9362
S39	5.9	0.3038	5.7	1.8291	0.0	0.0000	0.2760	2.4089
S40	5.8	0.1631	3.9	0.7200	1.4	0.0318	0.0815	0.9964
S41	1.1	0.2019	9.6	5.9719	4.6	1.0367	0.5137	7.7242
S42	0.7	0.1290	16.2	3.6132	2.1	0.1379	0.1264	4.0065
S43	1.1	0.0358	8.7	2.9161	3.5	0.1073	0.2996	3.3588
S44	0.7	0.1818	7.9	6.6803	0.0	0.0000	1.1054	7.9675
S45	0.8	0.1956	5.9	2.3759	10.8	2.1804	0.2250	4.9769
S46	0.9	0.1566	7.3	4.2965	12.4	0.2601	0.2287	4.9419
S47	1.1	0.1944	6.4	6.1561	4.3	0.3107	0.2449	6.9061
S48	0.9	0.3566	5.5	11.0254	7.3	2.9163	0.1478	14.4461

S49	0.8	0.2126	6.5	8.7367	4.2	1.8179	0.2919	11.0591
S50	1.7	0.6439	5.6	13.4963	4.9	8.8369	1.1510	24.1281
S51	1.7	0.1819	1.6	0.5246	6.9	1.6355	0.3832	2.7252
S52	1.1	0.1545	1.7	1.0451	3.5	1.1686	0.3034	2.6716
S53	1.9	0.3117	4.4	1.7780	6.5	0.5929	0.2687	2.9513
S54	1.8	0.3328	3.6	1.0626	5.0	1.4059	0.5922	3.4935
S55	1.7	0.2177	4.5	2.0990	5.0	0.5023	0.1931	3.0121
S56	1.6	0.2458	2.0	1.0600	3.0	1.1900	0.5129	3.0087
S57	2.7	0.3151	3.5	1.0635	5.1	0.7399	0.1797	2.2982
S58	4.2	0.3603	3.5	1.8270	4.1	0.6021	0.2243	3.0737
S59	3.0	0.2216	4.3	1.8669	2.8	0.5692	0.2341	2.7918
S60	5.2	0.7087	5.5	6.1292	6.2	1.2674	0.2253	8.3306
S61	2.7	0.3351	3.1	3.0888	1.9	1.1709	0.3052	4.9000
S62	4.6	0.5787	3.3	2.3893	5.3	2.2347	0.8232	6.0259
S63	2.1	0.6555	7.2	16.9813	6.3	7.5291	1.3639	26.5298
S64	1.0	0.1532	5.1	2.0268	8.0	0.5315	0.1842	2.8957
S65	1.9	0.6543	6.3	11.2022	6.0	7.3834	1.3445	20.5844
S66	1.5	0.205	5.5	2.4476	7	0.5343	0.2699	3.4568
S67	1.2	0.172	3	1.2318	3	0.2842	0.1017	1.7897
S68	1.8	0.109	1.5	0.585	3.9	0.655	0.1673	1.5163
S69	1.6	0.4238	4.5	3.4125	5.5	1.8108	0.5183	6.1654
S70	3.5	0.3334	2	0.6015	3.5	0.4183	0.2619	1.6151
S71	2.4	0.3538	1.8	1.47	6.9	3.6778	0.2516	5.7532
S72	4	0.29	3	1.2488	2.3	0.392	0.3054	2.2362
S73	4.6	0.1844	3.4	0.9841	3.5	0.3226	0.1266	1.6177

S74	4	0.3488	3	1.873	2.3	0.7564	0.3636	3.3418
S75	2.5	0.3636	3	2.3106	5.2	0.807	0.1805	3.6617
S76	2.7	0.6954	2.1	5.9115	3.3	1.8698	0.5907	9.0674
S77	2.1	0.2542	2.2	2.3852	3.7	1.1059	0.1729	3.9182
S78	3.8	0.2784	1.4	1.018	2.7	1.3459	0	2.6423
S79	3.4	1.105	2	0.9806	2.2	0.3403	0	2.4259
S80	2.7	0.1951	0.9	0.4482	3.8	1.3616	0.0098	2.0147
S81	1.9	0.063	3.7	1.6663	3.5	0.402	0	2.1313
S82	1.7	0.1093	0.5	0.971	1.7	2.288	0	3.3683
S83	1.3	0.4114	8.7	10.0835	4.6	1.2021	0.7801	12.4771
S84	1.3	0.5219	8.5	12.6749	6	1.9858	0.4976	15.6802
S85	0.9	0.502	5.4	12.049	5.2	3.3188	0.5666	16.4364
S86	1.5	0.4892	6.1	9.5526	4.2	1.8685	1.2852	13.1955
S87	1.5	0.673	9.2	15.4417	3.2	2.4719	1.0218	19.6084
S88	1.3	0.4128	4.6	9.2712	3.3	1.662	0.6531	11.9991
S89	1.2	0.0591	3.3	0.4303	5.1	0.2024	0.1103	0.8021
S90	1.9	0.0791	2.4	0.2203	3.3	0.1744	0.0703	0.5441
S91	1.7	0.0801	2.6	0.3044	4.2	0.1978	0.0897	0.672
S92	2.4	0.5116	3.4	2.4918	5.6	1.7149	0.5943	5.3126
S93	2.3	0.5635	4.5	3.0738	6.8	1.9934	0.6577	6.2884
S94	2.3	0.5345	4.0	2.7683	6.1	1.8934	0.6126	5.8088
S95	3.7	0.5658	2.6	3.4812	9.0	2.3772	0.9362	7.3604
S96	3.0	0.5562	2.9	4.3038	4.0	3.0061	0.9248	8.7909
S97	3.4	0.5550	2.8	4.0324	7.0	2.7634	0.9243	8.2751
S98	1.7	0.6512	7.3	11.1531	5.7	2.7838	0.6081	15.1962

S99	1	0.4762	6.7	9.6236	6.2	3.311	0.4946	13.9054
S100	0.7	0.3734	8.0	13.1412	5.2	1.5810	0.4321	15.5277
S101	1.1	0.3710	7.3	12.7340	5.4	1.7341	0.3010	15.1401
S102	0.9	0.3711	7.5	12.8734	5.3	1.6521	0.3503	15.2469
S103	2.1	0.2887	6.5	3.7655	4.0	1.1149	0.4328	5.6019
S104	2.6	0.4215	2.5	2.2811	4.0	1.0031	0.2972	4.0029
S105	2.3	0.4846	1.7	1.6174	6.0	3.2008	0.2636	5.5664
S122	2.2	0.6266	4.0	5.2241	5.9	3.4823	0.5564	9.8894
S123	2.5	0.3658	3.2	3.1249	4.3	1.6619	0.4311	5.5837
S106	3.5	0.2545	5.7	1.8004	2.0	0.0744	0.2123	2.3416
S107	3.2	0.2188	1.7	0.7755	7.0	2.1794	0.2228	3.3965
S108	3.4	0.3240	1.5	0.7848	5.3	1.5608	0.2796	2.9492
S124	3.8	0.3628	2.4	1.2220	6.8	2.0258	0.4296	4.0402
S125	4.0	0.2594	3.3	0.7342	4.5	1.2444	0.2350	2.4730
S109	3.3	0.8616	2.0	2.1907	12.6	5.2969	0.9047	9.2539
S110	3.7	0.9381	1.3	2.4732	5.0	3.2010	0.6596	7.2719
S111	4.4	0.9973	1.2	1.8531	8.1	4.7330	1.0110	8.5944
S126	4.0	0.8438	2.2	3.2026	7.5	4.7745	0.9019	9.7228
S127	4.5	1.0245	3.5	5.5048	6.6	2.6051	0.9000	10.0344
S112	5.0	0.9665	1.7	3.0234	8.0	4.2540	0.6511	8.8950
S113	4.8	1.1443	3.1	4.8542	4.2	1.4909	0.8244	8.3138
S114	4.5	0.6999	2.4	2.5452	4.1	2.8818	0.7341	6.8610
S128	4.4	0.7991	1.9	1.1915	5.8	3.4260	1.1609	6.5775
S129	4.2	1.1009	1.6	1.7224	4.1	3.2125	1.2051	7.2409
S115	1.3	0.4114	8.7	10.0835	4.6	1.2021	0.7801	12.4771

S116	1.3	0.5219	8.5	12.6749	6.0	1.9858	0.4976	15.6802
S117	0.9	0.5020	5.4	12.049	5.2	3.3188	0.5666	16.4364
S118	1.5	0.4892	6.1	9.5526	4.2	1.8685	1.2852	13.1955
S119	1.5	0.6730	9.2	15.4417	3.2	2.4719	1.0218	19.6084
S120	1.3	0.4128	4.6	9.2712	3.3	1.6620	0.6531	11.9991
S130	1.7	0.6512	7.3	11.1531	5.7	2.7838	0.6081	15.1962
S131	1.0	0.4762	6.7	9.6236	6.2	3.3110	0.4946	13.9054
S121	2.5	3.3462	7.6	36.2877	7.8	40.0700	6.8805	86.5844
S132	2.2	3.4735	6.6	24.853	7.7	42.1636	9.0873	79.5774

Table S15

Correlation analysis of pharmacognosy data with non-free oxalic acid and calcium oxalate crystal in MFCG

	Non-free oxalic acid	Calcium oxalate crystal
Growing age	0.641**	-0.02
Rhizome length	0.661**	0.235
Rhizome weight	0.421**	0.369*
Main root length	0.411**	0.089
Main root weight	0.340*	0.110
Lateral root length	-0.405**	-0.164
Lateral root weight	-0.285	-0.079
Fibrous root weight	0.040	0.237
Total weight	0.120	0.175

Table S16

Correlation analysis of pharmacognosy data with non-free oxalic acid and calcium oxalate crystal in GG

	Non-free oxalic acid	Calcium oxalate crystal
Rhizome length	0.310	0.211
Rhizome weight	0.079	-0.103
Main root length	0.285	0.358
Main root weight	0.067	0.067
Lateral root length	-0.455	-0.224
Lateral root weight	-0.152	-0.358
Fibrous root weight	0.673*	0.552
Total weight	0.115	0.018

Table S17

Correlation Analysis Results between the appearance traits of MFCG and organic acid content

	Growth year	Rhizome length	Rhizome weight	Main root length	Main root weight	Lateral root length	Lateral root weight	Fibrous root weight	Total weight
Growth year	1.000	0.785**	0.382	-0.135	0.339	-0.25	0.36	0.177	0.367
Oxalic acid	0.142	0.218	0.072	0.059	-0.079	-0.160	-0.076	0.055	-0.083
Malonic acid	-0.095	0.054	0.168	0.211	0.212	0.115	0.037	0.373	0.128
Fumaric acid	0.178	-0.004	0.232	-0.035	0.221	0.236	0.482*	-0.048	0.370
Succinic acid	-0.649**	-0.467*	-0.101	-0.037	0.056	0.409*	-0.011	0.181	0.002
Malic acid	0.317	0.271	0.227	0.151	0.295	-0.245	0.018	0.133	0.203
Cinnamic acid	-0.418*	-0.261	-0.361	-0.042	-0.318	0.135	-0.208	-0.078	-0.306
Citric acid	0.373	0.197	-0.192	-0.211	-0.208	-0.482*	-0.228	-0.365	-0.238
Palmitic acid	-0.041	0.075	-0.089	-0.308	-0.119	-0.319	-0.088	-0.194	-0.113
Total organic acid	-0.006	0.085	-0.039	-0.037	0.008	-0.169	-0.151	0.049	-0.090

Fumaric acid/ Total organic acid	0.195	0.002	0.208	0.020	0.198	0.159	0.426*	-0.050	0.325

Table S18

Correlation Analysis Results between the appearance traits of GG and organic acid content

	Rhizome length	Rhizome weight	Main root length	Main root weight	Lateral root length	Lateral root weight	Fibrous root weight	Total weight
Oxalic acid	-0.169	0.000	0.114	-0.109	-0.283	-0.173	0.282	0.036
Malonic acid	-0.014	0.100	0.200	0.018	-0.269	-0.036	0.227	0.064
Fumaric acid	-0.816**	-0.591	0.310	0.436	0.191	-0.482	-0.609*	0.218
Succinic acid	0.571	0.300	-0.059	-0.555	0.328	0.255	0.236	-0.473
Malic acid	-0.447	0.036	0.141	0.055	0.232	0.045	-0.055	0.227
Cinnamic acid	0.506	0.615*	-0.087	-0.515	0.432	0.533	0.342	-0.123
Citric acid	0.258	0.100	-0.150	-0.200	-0.009	0.191	0.136	-0.036
Palmitic acid	0.645*	0.773**	-0.191	-0.427	0.023	0.618*	0.555	-0.073
Total organic acid	0.300	0.436	0.014	-0.527	0.260	0.318	0.391	-0.182
Fumaric acid/ Total organic acid	-0.807**	-0.700*	0.360	0.591	0.223	-0.482	-0.745**	0.255

Table S19

Correlation Analysis Results between the appearance traits of MFCG and ginsenoside content

	Growth year	Rhizome length	Rhizome weight	Main root length	Main root weight	Lateral root length	Lateral root weight	Fibrous root weight	Total weight
Growth year	1.000	0.889**	0.698**	-0.396	0.047	0.194	0.458*	0.698**	0.458*
Rg1	0.279	0.179	0.173	-0.063	0.113	0.256	0.456*	0.379	0.107

Re	-0.078	-0.065	-0.447*	0.091	-0.397	0.011	-0.195	-0.223	-0.541*
Rb1	0.341	0.242	0.171	-0.171	-0.141	0.199	0.400	0.343	-0.005
Ro	0.039	0.162	0.188	0.142	0.171	0.549*	0.377	0.227	0.227
Rc	0.264	0.081	0.176	-0.213	0.048	0.248	0.441	0.317	0.105
Rb2	0.295	0.224	0.278	-0.169	0.042	0.123	0.417	0.353	0.260
Rd	0.140	-0.099	0.206	-0.105	0.126	-0.001	0.344	0.475*	0.188
SUM	0.271	0.163	0.116	-0.077	-0.042	0.171	0.415	0.313	0.021
PPD	0.357	0.212	0.242	-0.191	0.063	0.210	0.483*	0.415	0.188
PPT	0.233	0.165	-0.117	-0.024	-0.164	0.169	0.209	0.135	-0.242
PPD/PPT	0.403	0.249	0.595**	-0.368	0.202	0.269	0.630**	0.564**	0.648**
Rb1/Ro	0.155	0.009	-0.119	-0.291	-0.341	-0.271	-0.098	-0.012	-0.284

Note: SUM=Rg1+Re+Rb+Ro+Rc+Rb2+Rd, PPD=Rb1+Rb2+Rc+Rd, PPT=Rg1+Re。

Table S20

Correlation Analysis Results between the appearance traits of GG and ginsenoside content

	Growth year	Rhizome length	Rhizome weight	Main root length	Main root weight	Lateral root length	Lateral root weight	Fibrous root weight	Total weight
Growth year	1.000	0.707*	0.696*	0.000	0.696*	0.696*	0.696*	0.696*	0.696*
Rg1	0.174	-0.086	-0.055	0.103	0.200	0.491	0.309	-0.115	0.212
Re	0.174	-0.142	-0.079	-0.103	0.164	-0.030	0.006	0.345	0.127
Rb1	0.435	0.049	0.212	0.394	0.564	0.624	0.248	0.067	0.430
Ro	0.522	0.295	0.406	0.442	0.612	0.721*	0.370	0.115	0.442
Rc	0.696*	0.332	0.406	0.200	0.661*	0.479	0.309	0.515	0.552
Rb2	-0.696*	-0.382	-0.588	-0.200	-0.770**	-0.576	-0.503	-0.467	-0.697*

Rd	-0.348	-0.480	-0.345	0.091	-0.261	-0.467	-0.248	-0.212	-0.273
SUM	0.522	0.185	0.200	0.370	0.564	0.430	0.152	0.370	0.418
PPD	0.696*	0.308	0.467	0.333	0.758*	0.527	0.394	0.455	0.648*
PPT	0.174	-0.172	-0.067	0.079	0.273	0.115	0.055	0.200	0.224
PPD/PPT	0.696*	0.480	0.418	0.042	0.382	0.539	0.394	0.297	0.358
Rb1/Ro	-0.522	-0.332	-0.358	-0.527	-0.588	-0.624	-0.285	-0.176	-0.382

Note: SUM=Rg1+Re+Rb+Ro+Rc+Rb2+Rd, PPD=Rb1+Rb2+Rc+Rd, PPT=Rg1+Re。

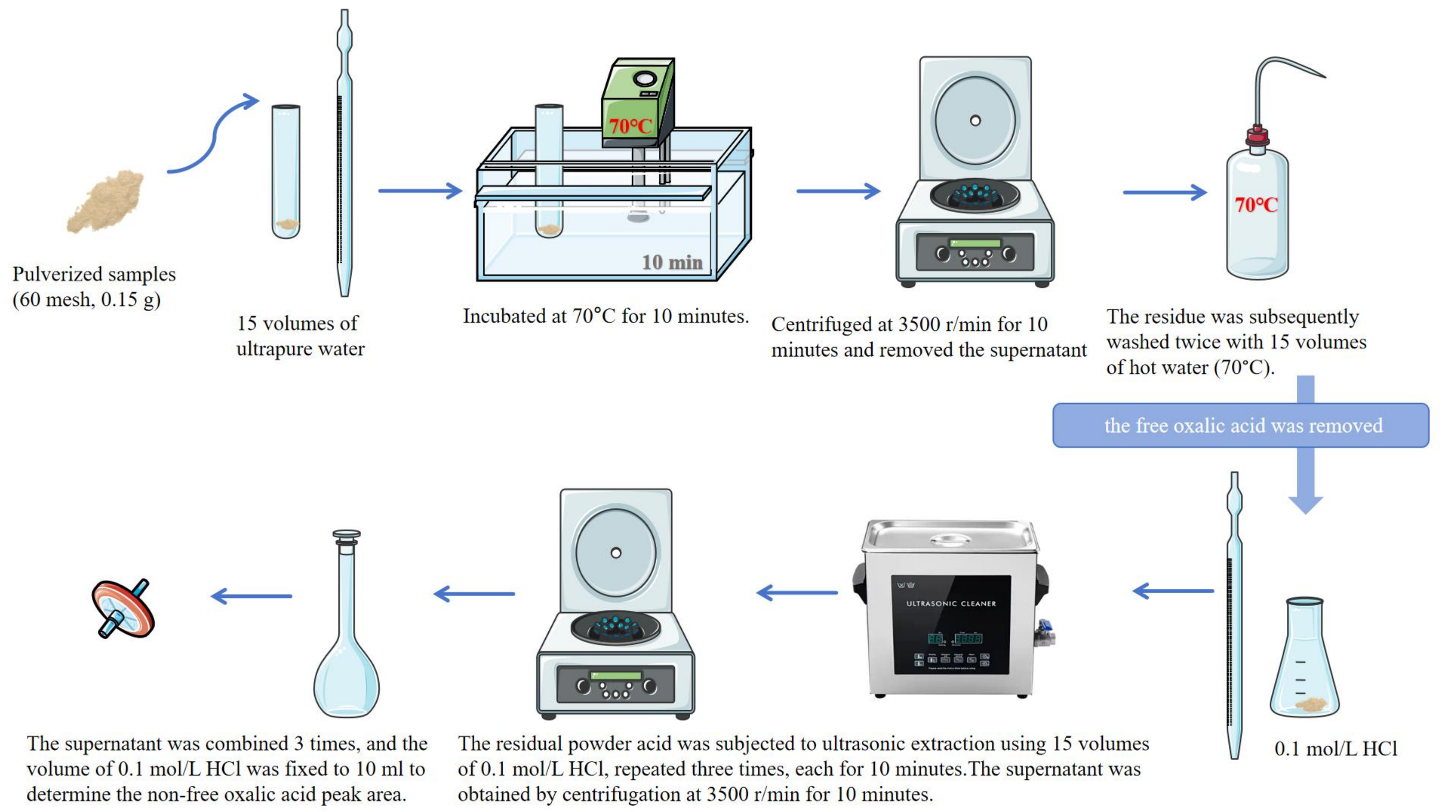

Figure S4 Sample solutions preparation for HPLC determination of non-free oxalic acid

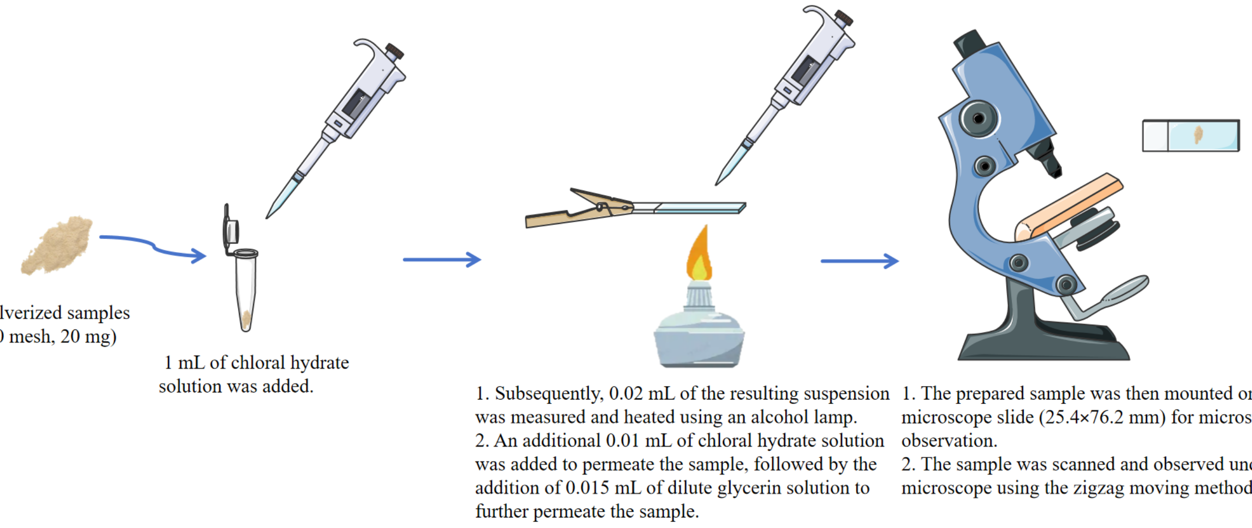

Figure S5 Sample solutions preparation for microscopic identification

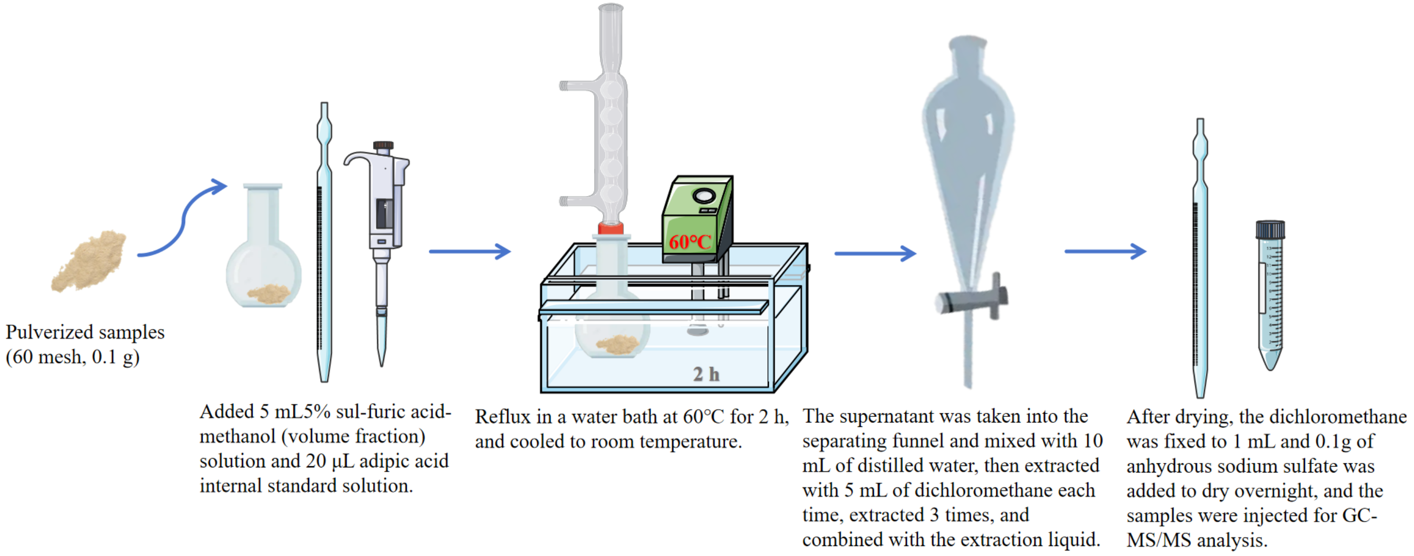

Figure S6 Sample solutions preparation for GC-MS/MS

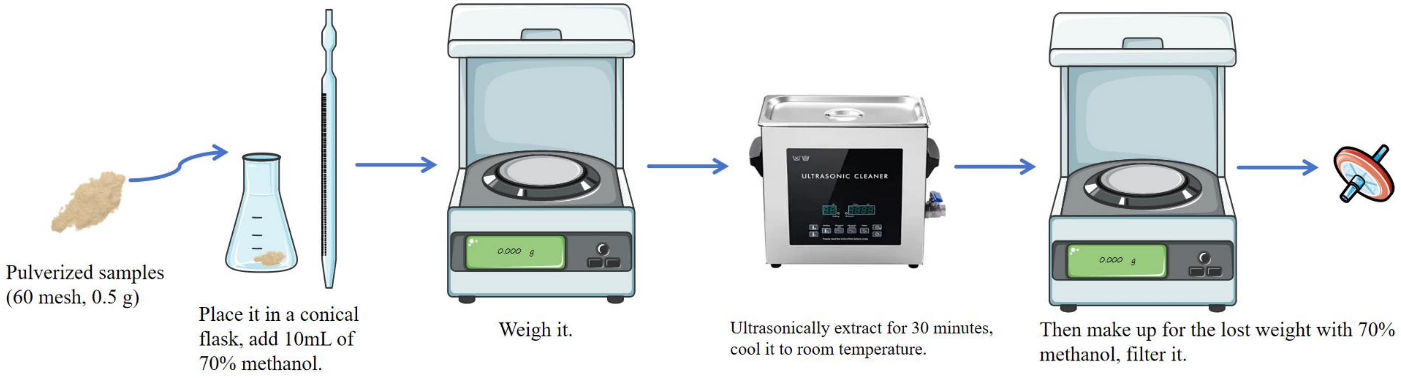

Figure S7 Sample solutions preparation for HPLC determination of ginsenosides
